# Functional Pore Accessibility and Surface Chemistry Govern Adsorption in Biomass-Derived Activated Carbons Under Real Aqueous Conditions

**DOI:** 10.3390/ma19132743

**Published:** 2026-06-26

**Authors:** Nelson de Jesús López-Acopa, Carlos Eduardo Santolalla-Vargas, María Patricia Torres-Magaña, David Salvador García-Zaleta, Juan Carlos Arévalo-Pérez, José Gilberto Torres-Torres, Areli Carrera-Lanestosa, Pedro García-Alamilla, Héctor Martínez-García, Zenaida Guerra-Que

**Affiliations:** 1 Laboratorio de Investigación 1 Área de Nanotecnología, Tecnológico Nacional de México-Instituto Tecnológico de Villahermosa (TECNM—I. T. Villahermosa), Km. 3.5 Carretera Villahermosa–Frontera, Cd. Industrial, Villahermosa 86010, Mexico; 2 Departamento de Biociencias e Ingeniería, Centro Interdisciplinario de Investigaciones y Estudios Sobre Medio Ambiente y Desarrollo (CIIEMAD), Instituto Politécnico Nacional, Ciudad de México 07340, Mexico; csantolallav@ipn.mx; 3 División Académica Multidisciplinaria de Jalpa de Méndez, Universidad Juárez Autónoma de Tabasco, Carr. Estatal Libre VHS-COM. Km. 27+000 S/N Ranch. Ribera Alta, Jalpa de Méndez 86205, Mexico; david.garcia@ujat.mx; 4 Laboratorio de Nanomateriales Catalíticos Aplicados al Desarrollo de Fuentes de Energía y Remediación Ambiental, Centro de Investigación de Ciencia y Tecnología Aplicada (CICTAT), DACB, Universidad Juárez Autónoma de Tabasco, Km. 1 Carretera Cunduacán-Jalpa de Méndez, Cunduacán 86690, Mexico; carlos.arevalo@ujat.mx (J.C.A.-P.);; 5 División Académica de Ciencias Agropecuarias (DACA), Universidad Juárez Autónoma de Tabasco (UJAT), Carret. Villahermosa-Teapa Km. 25, Ra. La Huasteca, Centro 86280, Mexico

**Keywords:** biomass-derived activated carbon, waste-derived carbonaceous adsorbents, functional pore accessibility, surface chemistry, structure–property–performance relationship, circular economy, adsorption mechanisms, Chemical Oxygen Demand (COD) removal, Dissolved Organic Matter (DOM), real aqueous systems

## Abstract

Biomass-derived activated carbons (ACs) are promising sustainable adsorbents for water polishing; however, their performance in real aqueous matrices cannot always be predicted from BET surface area alone. In this study, chemically activated biomass-derived carbonaceous adsorbents were prepared from Cocoa Pod Husk (CPH), Watermelon Peel (WP), and Pineapple Crown (PC) and evaluated for Chemical Oxygen Demand (COD) removal from real eutrophic lagoon water. The materials were characterized by N_2_ adsorption–desorption analysis, including BET surface area and BJH pore-size assessment, XRD, Raman spectroscopy, FTIR, UV–Vis diffuse reflectance spectroscopy, and pH_PZC_ analysis. Although all adsorbents exhibited low N_2_-BET surface areas, adsorption performance was governed by apparent functional pore accessibility inferred from adsorption behavior, pore size distribution, surface chemistry, structural disorder, electronic delocalization, and surface charge. Among the acid-activated samples, ACPCSA5 showed a narrow average pore size of 1.720 nm and achieved near-complete COD removal. Its superior performance was associated with oxygen-containing functional groups, partially developed sp^2^ carbon domains, lower optical band gap, BJH-derived pore architecture, and favorable surface charge at lagoon pH. The Microbial Regrowth Potential Index (MRPI) was introduced only as a conservative COD-based proxy, not as a validated biological indicator. Overall, this work demonstrates that adsorption in real-water matrices depends on accessible pore architecture and multifunctional surface chemistry rather than BET surface area alone.

## 1. Introduction

Water contamination driven by anthropogenic activities remains one of the most critical environmental challenges worldwide. Rapid urbanization, industrial discharges, agricultural runoff, and diffuse pollution sources introduce large quantities of organic and inorganic contaminants into aquatic ecosystems, deteriorating water quality and posing risks to environmental and human health [1,2,3,4,5,6,7]. In freshwater systems, these pressures are closely associated with eutrophication, a process driven by excessive inputs of nutrients and organic matter that stimulate algal growth, alter biogeochemical cycles, and disrupt ecological balance [1,8,9,10,11].

In eutrophic aquatic systems, the accumulation of dissolved and particulate organic matter plays a central role in water-quality degradation. Elevated organic substrate concentrations increase parameters such as Chemical Oxygen Demand (COD), Biological Oxygen Demand (BOD), and Total Organic Carbon (TOC), promoting microbial respiration and accelerating oxygen depletion in the water column [12,13]. Dissolved Organic Matter (DOM), which includes humic substances, microbial metabolites, algal-derived compounds, and anthropogenic organic residues, further influences nutrient availability, microbial activity, photochemical transformations, and the mobility of organic pollutants in natural waters [13,14,15,16]. Therefore, reducing the residual oxidizable organic load is an important component of strategies aimed at mitigating eutrophication and improving water quality.

These processes are particularly relevant in tropical urban lagoons, where high temperatures, intense solar radiation, shallow-water conditions, and recurrent anthropogenic inputs can intensify biological productivity and organic matter decomposition [9,12]. Surface runoff from urbanized catchments, leakage from sewage networks, urban drainage, and recreational activities may introduce nutrients, suspended particles, and organic residues into lagoon systems, contributing to sustained eutrophication pressure [15,17]. In addition, seasonal variations in precipitation, water residence time, and pollutant inputs can substantially modify the concentration and composition of DOM, leading to fluctuating organic substrate availability between dry and rainy periods [9,11,17]. Understanding these matrix-dependent variations is essential for designing remediation strategies suitable for tropical urban water bodies.

Conventional remediation approaches for eutrophic aquatic systems typically rely on nutrient management, wastewater treatment infrastructure, pathogen control, and ecological restoration. Although large-scale sanitation and wastewater treatment upgrades have improved water quality in several historically polluted systems [18,19,20], such infrastructure-intensive approaches are not always feasible for smaller urban lagoons, particularly in developing or rapidly urbanizing regions where economic, spatial, and operational constraints may limit implementation [18]. Moreover, conventional treatment technologies such as coagulation–flocculation, membrane filtration, ion exchange, and advanced oxidation processes may involve high operational costs, complex infrastructure, incomplete DOM removal, or the generation of secondary pollutants [21,22]. These limitations have encouraged the development of complementary, cost-effective, and environmentally sustainable strategies for reducing residual organic loads in aquatic systems [8,18,23].

Among these strategies, adsorption has emerged as a versatile approach for removing dissolved organic compounds and other contaminants from water because of its operational simplicity, flexibility, and compatibility with a wide range of adsorbent materials [3,4,18]. In the context of eutrophic water bodies, adsorption should not be considered a replacement for conventional treatment or watershed-level management, but rather as a complementary polishing strategy aimed at decreasing residual oxidizable organic substrates. Adsorption performance depends not only on surface area, but also on the accessibility of adsorption sites, pore architecture, surface functional groups, and the physicochemical interactions established between the adsorbent and the heterogeneous organic matrix, including electrostatic interactions, hydrogen bonding, hydrophobic effects, and π–π interactions [8,24,25,26].

Nanostructured carbon materials have attracted considerable attention as adsorbents for water purification because their adsorption performance can be tuned through pore architecture, surface chemistry, aromatic domains, and structural disorder [8,21]. Within this broader field, biomass-derived carbonaceous materials offer a sustainable route for producing low-cost adsorbents from lignocellulosic residues. Agro-industrial wastes composed mainly of cellulose, hemicellulose, and lignin are abundant, renewable precursors that can be transformed into functional carbon-based adsorbents through activation processes [2,3,6,24]. These materials may exhibit accessible pore networks, oxygenated surface functionalities, and disordered aromatic carbon domains that favor interactions with organic contaminants [3,18,23,25]. Thus, converting agro-industrial residues into adsorbents provides a dual environmental benefit: improving water-treatment options while supporting circular-economy strategies through the valorization of underutilized biomass.

For the present work, the investigated materials are described as biomass-derived chemically activated carbonaceous adsorbents. Their performance is evaluated in relation to precursor composition, activation chemistry, surface functionality, disordered sp^2^ carbon domains, and pore-related adsorption behavior inferred from N_2_ adsorption–desorption and batch adsorption results rather than from high BET surface area alone. In this context, functional pore accessibility is discussed as an apparent, performance-based descriptor inferred from adsorption behavior under real aqueous conditions, not as a direct morphological feature.

In tropical regions such as Mexico, fruit-derived lignocellulosic residues, including CPH, WP, and PC, are abundant and remain underutilized despite their potential as precursors for carbonaceous adsorbents [23,26,27,28,29]. Previous studies have shown that biomass-derived ACs and related carbonaceous materials can remove diverse contaminants from water, including dyes, pharmaceuticals, heavy metals, and natural organic matter [18,26,30,31,32]. Our previous work demonstrated that CPH-derived AC can effectively adsorb methylene blue under controlled single-solute conditions, providing insight into adsorption kinetics, isotherm behavior, and the role of activation chemistry [26].

However, the adsorption performance observed in synthetic model solutions does not necessarily translate directly to natural aquatic environments. Real water matrices contain heterogeneous DOM fractions with different molecular weights, functional groups, origins, and affinities for adsorbent surfaces [7,12,13,33,34]. These components may compete for adsorption sites, promote matrix-dependent interactions, and reduce the predictive value of conventional structure–property descriptors obtained under simplified laboratory conditions. Although biomass-derived carbonaceous adsorbents have been widely investigated for water treatment, relatively few studies have evaluated their performance directly in natural eutrophic lagoon water, particularly under seasonal hydrological variability.

In this context, Laguna de las Ilusiones, located in Villahermosa, Mexico, was selected as a representative eutrophized tropical urban lagoon to evaluate adsorption behavior under real aqueous conditions. This system provides a complex and temporally variable matrix for assessing whether waste-derived chemically activated carbonaceous adsorbents can reduce oxidizable organic load beyond simplified model systems. Importantly, the study does not aim to present COD removal as direct biological evidence of microbial regrowth suppression, but as a physicochemical indicator of residual oxidizable organic matter.

Therefore, this work aims to elucidate how precursor composition, activation route, surface chemistry, and apparent functional pore accessibility, as inferred from adsorption behavior, govern the adsorption performance of waste-derived chemically activated carbonaceous adsorbents in real lagoon water.

By integrating structural characterization with COD removal performance and a conservative COD-derived framework for residual organic load evaluation, this study establishes a structure–property–performance relationship that supports predictive adsorbent design beyond conventional BET surface area descriptors.

## 2. Materials and Methods

### 2.1. Study Area and Water Sampling

Water samples were collected from Laguna de las Ilusiones, an urban tropical lagoon located in Villahermosa, Tabasco, Mexico (17°59′ N, 92°56′ W). The lagoon covers approximately 222 hectares and is part of a protected natural area (Área Natural Protegida Laguna de las Ilusiones, designated in 1995). It provides key ecological and social functions, including habitat provision, microclimate regulation, and recreational use [35].

The lagoon is located within the Grijalva River basin, under a humid tropical climate characterized by high annual precipitation (~2000–2500 mm) and average temperatures ranging from 24 to 38 °C. Despite its ecological importance, the system is increasingly impacted by urbanization, insufficient wastewater treatment, and diffuse pollutant inputs from surrounding residential and commercial areas [36,37,38].

**Figure 1:**
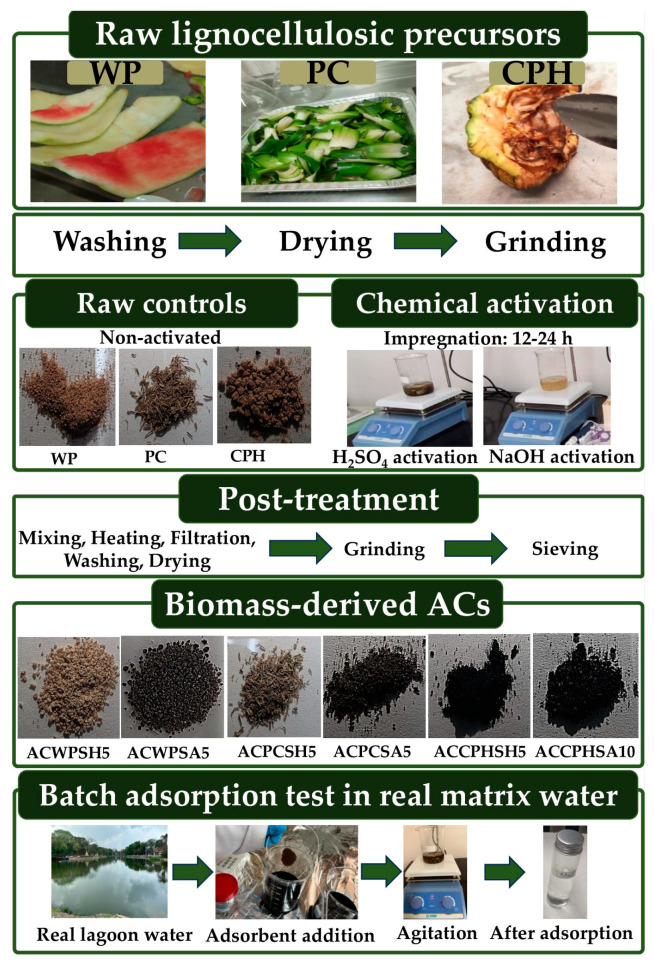
Experimental workflow for the preparation, post-treatment, and evaluation of waste-derived ACs from lignocellulosic wastes.

Sampling campaigns were conducted during two contrasting hydrological periods, representative of dry and rainy seasons, to evaluate seasonal variability. Surface water samples were collected from multiple points within the lagoon, including sites previously identified as high-pollution zones based on local environmental monitoring reports (Figure 1).

Samples were collected using pre-cleaned polyethylene containers, transported under refrigerated conditions (4 °C), and analyzed within 24 h to minimize physicochemical alterations.

The selected sampling sites were used to assess the adsorption performance of Biomass-derived ACs derived from locally available lignocellulosic waste, to propose a low-cost and sustainable strategy for the remediation of urban tropical water systems.

### 2.2. Raw Materials and Precursor Preparation

Three lignocellulosic residues abundant in tropical regions were selected as ACs precursors: CPH, WP, and PC. These agro-industrial wastes are widely available and have been extensively reported as suitable feedstocks for the production of carbon-based adsorbents due to their lignin-rich structure and high carbon content [25,39].

The collected biomass was initially washed thoroughly with deionized water to remove adhered dust and surface impurities. Subsequently, the materials were air-dried under sunlight for three days on a flat, open surface, followed by oven drying at 100 °C for 48 h (Riossa, Mexico City, Mexico) to ensure complete moisture removal. The dried biomass was then ground and sieved using a mechanical sieve shaker to obtain a homogeneous particle size below 0.25 mm (mesh size 60–80), suitable for subsequent thermal and chemical treatments.

The selected particle size range was chosen to minimize internal diffusion limitations and ensure reproducible adsorption kinetics.

### 2.3. Synthesis of Biomass-Derived Activated Carbons: Chemical Activation

Activated materials were synthesized via chemical activation following a protocol adapted from previous work on CPH-derived ACs [26]. The process involved chemical impregnation of the lignocellulosic precursors without subsequent thermal carbonization.

The prepared biomass samples (CPH, WP, and PC) were subjected to chemical activation using either sulfuric acid (H_2_SO_4_; Sigma-Aldrich, St. Louis, MO, USA) or sodium hydroxide (NaOH; Sigma-Aldrich, St. Louis, MO, USA) solutions. The delignification and activation treatments were conducted using a solid-to-liquid ratio of 1:10 (*w*/*v*) at room temperature for 12 h. For alkaline activation, biomass samples were immersed in NaOH solutions at a concentration of 5 M (100 g L^−1^), yielding materials denoted as ACCPHSH5, ACWPSH5, and ACPCSH5. For acidic activation, samples were treated with H_2_SO_4_ at concentrations of 5 M and 10 M, producing ACCPHSA10, ACWPSA5, and ACPCSA5.

After chemical treatment, all samples were vacuum-filtered and extensively washed with deionized water until a neutral pH was reached to ensure the removal of residual activating agents and soluble by-products. A final rinsing step with acetone was applied to facilitate drying and remove residual organics. The resulting solids were oven-dried at 80 °C until constant weight (typically 24 h) and subsequently stored for further characterization and adsorption experiments.

In this work, the term “Activated Carbon” is used in a non-conventional sense to describe chemically activated, biomass-derived carbonaceous adsorbents obtained from lignocellulosic residues through acid or alkaline treatment. This terminology is consistent with previous reports on biomass-derived ACs prepared through chemical activation routes, including CPH, WP, PC, and other agro-industrial waste-derived adsorbents [26,40,41,42,43]. However, these materials should not be considered equivalent to conventional commercial ACs produced by full thermal carbonization followed by physical activation, which typically exhibit highly developed porosity and very high BET surface areas. Rather, they are referred to here as chemically activated, waste-derived carbonaceous materials with modified surface chemistry, structural features, and adsorption functionality. This distinction is important because the adsorption behavior evaluated in this study occurs under real lagoon-water conditions, where DOM removal may depend not only on BET surface area, but also on functional pore accessibility, oxygenated surface groups, electrostatic interactions, hydrogen bonding, and heterogeneous carbonaceous domains.

Because these materials were not subjected to conventional high-temperature carbonization, their long-term aqueous stability and potential release of soluble organic or inorganic fractions require further evaluation. Future work should include leaching assays, TOC release measurements, pH and conductivity monitoring after aqueous contact, repeated washing cycles, and regeneration studies to quantify the stability, reusability, and practical applicability of these non-conventional chemically activated carbonaceous adsorbents under real aqueous conditions.

### 2.4. Physicochemical Characterization of Waste-Derived ACs

The synthesized materials were characterized using a combination of analytical techniques to evaluate their structural, textural, and chemical properties.

N_2_ adsorption–desorption isotherm

Textural properties were determined by N_2_ adsorption–desorption isotherms. The specific surface area, pore volume, and pore size distribution of CPH, PC, WP, ACCPHSH5, ACWPSH5, ACPCSH5, ACCPHSA10, ACWPSA5, and ACPCSA5 were measured via N_2_ physisorption using a Micromeritics TriStar II 3020 (Micromeritics Instrument Corp., Norcross, GA, USA) surface area analyzer at 77 K (−196 °C). Before analysis, approximately 0.1 g of each sample was degassed at 300 °C for 3 h to remove physically adsorbed species and moisture.

The adsorption–desorption isotherms were analyzed using BET model to determine the specific surface area (S_BET_) (Micromeritics Instrument Corporation, Norcross, GA, USA). The total pore volume (P_V_) and average pore diameter (P_S_) were calculated using the Barrett–Joyner–Halenda (BJH) method based on the desorption branch, as implemented in the ASAP 2020 software package [41,44].

These parameters are critical to understanding adsorption performance, as surface area and pore structure directly influence accessibility of active sites and mass transfer phenomena in lignocellulosic-derived carbons [25].

Raman Spectroscopy

Raman spectroscopy was employed to evaluate the structural order and defect density of the AC materials. Spectra were recorded using a HORIBA Xplora Plus Raman microscope (HORIBA France SAS, Palaiseau, France) equipped with a He–Ne laser (λ = 632.8 nm, 16 mW) and a thermoelectrically cooled CCD detector. The spectral resolution ranged from ±0.2 to ±0.5 cm^−1^.

Measurements were performed using 10×, 50×, and 100× objectives to ensure appropriate signal acquisition across heterogeneous sample regions.

The characteristic D (~1350 cm^−1^) and G (~1580 cm^−1^) bands were analyzed to assess structural disorder and graphitic domain development. The intensity ratio (I_D_/I_G_) was calculated to estimate defect density and degree of graphitization in the biomass-derived ACs, as widely established for carbon-based materials [45]. These structural features are directly related to the presence of aromatic domains and oxygen-containing functional groups, which influence adsorption behavior in biomass-derived ACs [46].

X-Ray Diffraction (XRD)

Structural properties and degree of ordering of the AC materials were analyzed by XRD. Measurements were performed using a Bruker D2 PHASER diffractometer (Bruker AXS SE, Karlsruhe, Germany) equipped with Co Kα radiation (λ = 0.179 nm). Diffraction patterns were recorded over a 2θ range of 20–80° with a total scan time of 660 s.

Phase identification and pattern analysis were conducted using JADE 6 software and the corresponding reference database. Particular attention was given to the broad diffraction features associated with partially ordered or turbostratic carbon structures typically observed in biomass-derived ACs, whose structural properties strongly depend on precursor composition and activation conditions [25,44].

The apparent crystallite size (D) was estimated using the Scherrer equation (Equation (1)):

(1) $$D=\frac{0.90\lambda }{\beta cos\theta }$$

where D is the crystallite size, λ is the X-ray wavelength, β is the full width at half maximum (FWHM) of the diffraction peak, and θ is the Bragg angle. This approach is commonly applied to estimate the size of ordered domains in partially disordered carbonaceous materials derived from lignocellulosic biomass [8].

Ultraviolet–Visible Spectroscopy with Diffuse Reflectance (UV-Vis DR)

UV–Vis DR spectra were recorded in the range of 200–900 nm using a Varian Cary 3000 spectrophotometer (Varian Inc., Palo Alto, CA, USA) equipped with an integrating sphere accessory, operating at room temperature. Barium sulfate (BaSO_4_) was used as the reference material to establish 100% reflectance.

The reflectance data (R) were transformed using the Kubelka–Munk function (Equation (2)):

(2) $$F(R)=\frac{{\left(1-R\right)}^{2}}{2R}$$

The transformed spectra were used to analyze optical absorption features associated with electronic transitions in the carbon materials. The apparent optical band gap was estimated from Tauc plots derived from the Kubelka–Munk function, assuming indirect allowed transitions [47,48].

The optical properties were further correlated with structural characteristics obtained from Raman spectroscopy and textural properties derived from BET analysis.

Fourier Transform Infrared Spectroscopy (FTIR)

FTIR was performed using a PerkinElmer Frontier spectrophotometer (PerkinElmer Inc., Waltham, MA, USA) equipped with a diamond attenuated total reflectance (ATR) accessory. Spectra were recorded in the wavenumber range of 400–4000 cm^−1^ with a resolution of 4 cm^−1^, averaging 32 scans per spectrum.

Samples included untreated CPH, WP, and PC, and the corresponding chemically activated biomass-derived carbonaceous adsorbents to evaluate chemical transformations induced by acid and alkaline activation.

All spectra were subjected to baseline correction, smoothing, and normalization using PerkinElmer Spectrum software, version 10.5.4. Measurements were conducted in triplicate to ensure reproducibility.

The spectra were analyzed to identify functional groups and surface chemical features associated with lignocellulosic components and their transformation into oxygen-containing functionalities in the biomass-derived AC materials, which are known to depend on precursor composition and activation processes [6,25]. These functional groups play a key role in the adsorption behavior of carbon-based materials [8,46].

Point of Zero Charge (pH_PZC_) determination

The pH_PZC_ of the adsorbents was determined using the pH drift method to evaluate surface charge behavior. Briefly, 50 mL of 0.01 M NaCl solution was placed in a series of closed Erlenmeyer flasks, and the initial pH (pH_i_) was adjusted between 2.0 and 12.0 using 0.1 M HCl or 0.1 M NaOH.

A fixed mass of adsorbent (0.05 g) was added to each solution, and the suspensions were agitated at room temperature for 24 h to ensure equilibrium. The final pH (pH_f_) was then recorded, and the difference (ΔpH = pH_f_ − pH_i_) was plotted as a function of pH_i_. The pH_PZC_ was determined as the point at which ΔpH = 0, corresponding to the condition where the adsorbent surface exhibits no net electrical charge.

This method is widely applied for lignocellulosic and carbonaceous adsorbents to elucidate surface charge properties and their influence on adsorption mechanisms, particularly in systems involving DOM and charged species [24,49]. The surface charge behavior governed by pH_PZC_ plays a critical role in adsorption performance, as it controls electrostatic interactions between adsorbents and pollutants in aqueous systems [23].

### 2.5. Adsorption Experiments

Batch adsorption experiments were conducted using real lagoon water collected during seasonal sampling campaigns. A fixed adsorbent dosage of 4 g L^−1^ was applied in all experiments to ensure comparability among biomass-derived materials.

Experiments were carried out in 0.150 L of real water at an initial pH of approximately 6 under constant agitation at 250 rpm and room temperature. Samples were collected at predefined contact times ranging from 0 to 180 min, considering that adsorption performance is strongly influenced by parameters such as contact time, pH, and adsorbent dosage [23].

After each adsorption interval, suspensions were vacuum-filtered using 1.2 µm glass microfiber filters to remove residual solids before analysis.

The experimental design was intended as a screening-level evaluation rather than detailed kinetic modeling, aiming to compare the adsorption performance of the materials under realistic environmental conditions. Adsorption is widely recognized as an efficient and flexible method for contaminant removal in aqueous systems [24], and its performance depends on both adsorbent properties and water matrix composition [50].

The adsorption capacity at equilibrium, q_e_ (mg g^−1^), was calculated using Equation (3):

(3) $$q_{e}=\frac{{(C}_{0}-C_{e})V}{W}$$

where C_0_ and C_e_ (mg L^−1^) are the initial and equilibrium COD concentrations, respectively, V (L) is the solution volume, and W (g) is the mass of adsorbent.

The COD removal efficiency (%) was calculated as:

(4) $$R_{\%}=\frac{{(C}_{0}-C_{e})}{C_{0}}\times 100$$

### 2.6. Adsorption Kinetics

Adsorption kinetics were evaluated using Pseudo-First-Order (PFO) and Pseudo-Second-Order (PSO) models. Detailed equations, fitting methodology, nonlinear regression procedures, and complete kinetic parameters are provided in Section S1 of the Supplementary Information.

### 2.7. Analytical Determination of Organic Load

COD was determined using the standard dichromate digestion method (5220D). Analyses were performed using commercially available HACH digestion vials containing potassium dichromate (K_2_Cr_2_O_7_) and sulfuric acid (H_2_SO_4_) reagents, with a measurement range of 0–2500 mg L^−1^.

For each analysis, 2 mL of sample or blank was added to the digestion vials. Calibration was performed using potassium hydrogen phthalate standards covering the relevant COD concentration range. The vials were digested at 150 °C for 2 h, then measured spectrophotometrically at 600 nm using an HI801 Iris spectrophotometer (Hanna Instruments, Woonsocket, RI, USA).

All analyses were conducted in triplicate to ensure reproducibility. To minimize potential interferences in COD measurements associated with suspended carbon fines and chloride content, samples were subjected to filtration before analysis. However, it is acknowledged that residual fine particles and matrix complexity in real water samples may influence COD readings. Therefore, COD results should be interpreted as operational indicators of bulk organic load rather than absolute quantification of dissolved organic carbon.

COD was used as a bulk indicator of organic load in the lagoon water, as widely reported for evaluating the presence of organic contaminants in aqueous systems [23,24].

To avoid overinterpretation of the treatment mechanism, COD removal was considered the primary measured engineering response. In contrast, adsorption was used as the mechanistic framework supported by the physicochemical and structure–function characterization of the adsorbents, including FTIR, pH_PZC_, Raman spectroscopy, XRD, UV-Vis DR, and N_2_ adsorption–desorption analysis. Because the experiments were conducted under real static-water conditions using a complex lagoon matrix, the observed COD decrease may not be attributed exclusively to molecular adsorption. Secondary processes such as particle aggregation, matrix-induced association of DOM, or flocculation-like effects promoted by adsorbent–water interactions cannot be fully excluded. Therefore, this limitation has been explicitly acknowledged. Future work should include particle-size tracking, time-resolved zeta-potential measurements, turbidity monitoring, dissolved organic carbon fractionation, and filtration-control experiments to more quantitatively distinguish adsorption, aggregation, and possible secondary flocculation-like contributions to the overall COD removal response.

Figure 1 summarizes the overall experimental workflow, including precursor preparation, chemical activation, post-treatment, adsorbent production, and batch adsorption testing using real lagoon water.

### 2.8. Conceptual Microbial Regrowth Potential Index (MRPI) Framework

To interpret adsorption performance within a lagoon-management context, a conceptual MRPI framework was introduced. In this study, MRPI should be interpreted strictly as a COD-derived, substrate-based proxy linking residual oxidizable organic load to potential substrate availability. It is not intended to represent a validated microbiological regrowth index, nor does it demonstrate microbial inactivation, pathogen removal, microbial community shifts, or biological regrowth suppression.

COD was used because it provides an integrated measure of chemically oxidizable organic matter in complex aqueous matrices. However, COD does not distinguish between biodegradable and non-biodegradable fractions, nor between high- and low-molecular-weight dissolved organic compounds. Therefore, even when residual COD is low, biodegradable low-molecular-weight organic fractions may remain and could potentially support bacterial regrowth under favorable post-treatment or storage conditions.

Within this conservative framework, adsorption performance was evaluated not only in terms of COD removal efficiency, but also in terms of the extent to which adsorption reduces the residual oxidizable organic pool that may serve as substrate for heterotrophic microbial regrowth. Since COD is widely used as an indicator of organic contamination in water systems [23,24], reductions in COD were interpreted here as indicating a decrease in bulk organic-load availability, rather than as direct evidence of biological stability.

The MRPI was defined as:

(5) $$MRPI=\frac{{COD}_{f}}{{COD}_{i}}$$

where COD_i_ and COD_f_ represent the initial and final COD (mg L^−1^), respectively.

MRPI is used here as a COD-derived conceptual indicator of residual oxidizable organic load and is only indirectly related to substrate availability for heterotrophic microorganisms. It should not be interpreted as a direct measurement of Assimilable Organic Carbon (AOC), Biodegradable Dissolved Organic Carbon (BDOC), microbial abundance, or microbial regrowth.

By integrating MRPI into the analysis, the framework extends beyond removal efficiency to include:

- Post-treatment water stability.
- Risk of microbial recolonization.
- Long-term system performance.

The corresponding reduction in regrowth potential was estimated as:

(6) Regrowth Potential Reduction=1−MRPI

This conceptual framework assumes that adsorption processes reduce the pool of organic compounds in solution, which are commonly removed through adsorption-based treatments [51], thereby potentially limiting microbial regrowth in eutrophic systems.

### 2.9. Integrated Techno-Economic Framework for Adsorption-Based COD Removal in Real Water Matrices

To evaluate the practical feasibility of waste-derived chemically activated carbonaceous adsorbents for real-water treatment, an integrated techno-economic framework was developed at a laboratory scale. This analysis is intended as a preliminary techno-economic screening estimate, rather than a complete process cost assessment. The assessment does not include adsorbent regeneration, adsorbent recovery, hydraulic operation, sludge management, capital expenditure, long-term operation, or full-scale process integration. Therefore, the reported values should be interpreted as indicative laboratory-scale estimates.

Importantly, the term “low-cost” in this study refers primarily to the availability and valorization potential of lignocellulosic waste precursors, not to the total treatment cost per cubic meter under the single-use experimental conditions. This distinction is necessary because the estimated treatment cost depends strongly on adsorbent dosage, production cost, removal efficiency, regeneration potential, and process scale.

The techno-economic indicators were calculated from adsorbent dosage, estimated adsorbent production cost, treatment volume, COD removal efficiency, and annual treated volume. The full set of equations used to estimate treatment cost per unit volume, cost per kg COD removed, and annual COD abatement is provided in Section S2 of the Supplementary Information.

## 3. Results and Discussion

### 3.1. Textural and Surface Properties

#### 3.1.1. BET Surface Area Analysis: N_2_ Adsorption–Desorption Isotherm

Table 1 presents the textural parameters of six adsorbents, determined from the N_2_ adsorption–desorption isotherms using the BET method. The BET Specific Surface Area (S_BET_), micropore volume, total/small pore volume, and average pore size were evaluated to assess the effect of chemical activation on pore development and structural evolution. Overall, the materials exhibited low S_BET_ values, ranging from 0.5807 to 1.1260 m^2^ g^−1^, indicating limited surface area in the raw precursors and modest porosity in the chemically treated samples. However, the average pore size values of CPH, WP, PC, ACWPSA5, and ACPCSA5 ranged from 0.377 to 1.720 nm, placing these materials within the microporous domain according to the IUPAC classification, where microporous carbons are defined as materials with pore sizes below 2 nm, mesoporous carbons between 2 and 50 nm, and macroporous carbons above 50 nm. This indicates that these adsorbents can be described as nanoporous carbonaceous materials with a predominantly microporous character. Similar classifications have been reported for ACs and biomass-derived carbons, where micropore filling is associated with pore widths below 2.0 nm, while the coexistence of mesopores may be inferred from broader pore size distributions or hysteresis behavior in N_2_ isotherms [24,44].

Among the activated samples, ACPCSA5 exhibited an average pore size of 1.720 nm, confirming the development of microporous domains after acid treatment. This is particularly relevant because micropores provide high-energy adsorption sites, whereas narrow mesopores can facilitate molecular diffusion toward internal adsorption domains. In this sense, the textural behavior of these materials should not be interpreted only in terms of total BET surface area, but also in terms of pore accessibility, pore size distribution, and surface chemistry. Previous studies on biomass-derived ACs have shown that adsorption performance is controlled by a combination of internal pore structure, surface characteristics, pore volume, precursor chemistry, functional groups, and activation conditions [24].

ACCPHSH5 showed the highest apparent micropore volume (0.001692 cm^3^ g^−1^), together with one of the highest S_BET_ values among the activated samples (0.8299 m^2^ g^−1^). Nevertheless, the discrepancy between the BET-derived average pore size (~3.15 nm) and the MP-derived pore size (~0.315 nm) suggests that this material does not possess a single homogeneous pore population, but rather a heterogeneous pore network containing both microporous and small mesoporous domains. This interpretation is consistent with reports on cocoa pod husk-derived biochars, where N_2_ adsorption isotherms revealed type I microporous behavior associated with pore widths <2.0 nm, together with type IV/hysteresis features related to narrow mesopores [44]. In addition, AC derived from lignocellulosic residues has also been reported to contain coexisting micro- and mesoporous structures after chemical activation [52].

Despite the relatively higher micropore volume of ACCPHSH5, this material did not exhibit the highest adsorption performance in real lagoon water. This result reinforces that, under complex aqueous matrices, adsorption is not governed exclusively by S_BET_ or total micropore volume. Instead, the effective removal of COD appears to depend on the accessibility of the pore network, the organization of micro/mesoporous domains, and the availability of surface functional groups capable of interacting with DOM. Therefore, the most efficient adsorbent was not necessarily the one with the largest surface area or micropore volume, but the one with the most accessible and functionally optimized pore architecture. This interpretation agrees with recent reports indicating that biomass-derived carbon adsorbents with modest surface areas can still show relevant adsorption performance when a suitable porous structure and oxygen-containing surface-active sites are present [8,18,19,24,25,44,46,53].

#### 3.1.2. Structural Characterization by X-Ray Diffraction and Raman Spectroscopy

XRD and Raman spectroscopy were analyzed together because both techniques provide complementary information on the structural organization of the carbonaceous matrix. XRD was used to evaluate the turbostratic character and apparent coherent domain size, whereas Raman spectroscopy provided information on defect density, sp^2^-domain organization, and aromatic-domain reorganization. Therefore, this subsection focuses on the structural features of the waste-derived chemically activated carbonaceous adsorbents, while FTIR-based surface chemistry is discussed separately in Section 3.1.4.

**Figure 2:**
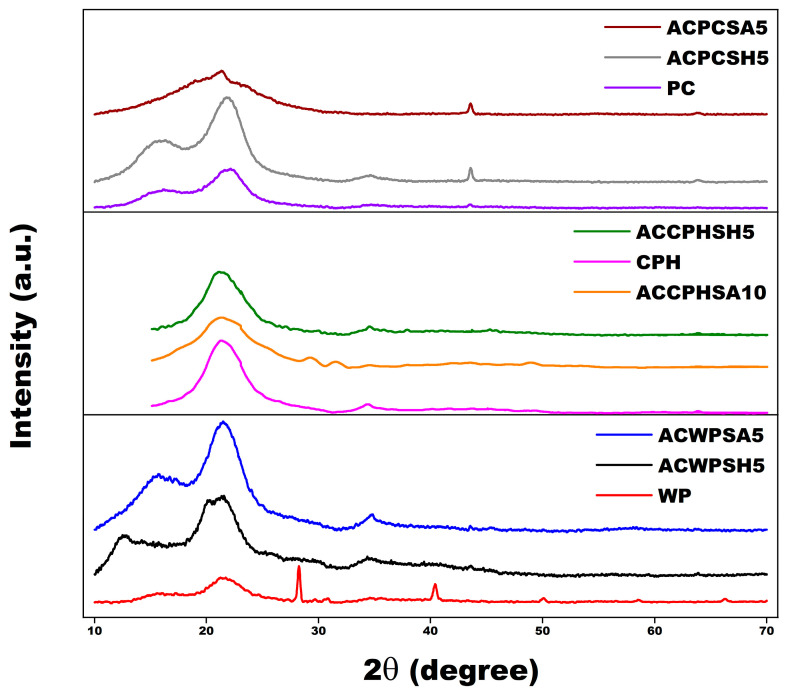
XRD patterns of Biomass-derived ACs synthesized from CPH, WP, and PC.

The XRD patterns of all samples (Figure 2) exhibit a broad diffraction band centered at approximately 21–22°, corresponding to the (002) plane of turbostratic carbon, along with a less defined feature near ~43° associated with the (100) plane. The absence of sharp diffraction peaks confirms the predominantly amorphous and disordered nature of the biomass-derived carbonaceous materials [54,55].

The apparent coherent domain size, estimated from the (002) band using the Scherrer equation, ranged from 1.16 to 2.59 nm (Table S1). Samples such as CPH (2.59 nm) and ACCPHSH5 (2.46 nm) exhibited relatively larger ordered domains, suggesting a higher degree of local stacking coherence. In contrast, ACPCSA5 (1.16 nm) and ACCPHSA10 (1.97 nm) showed smaller apparent domain sizes, reflecting increased structural disorder and reduced stacking coherence.

These differences are associated with the chemical activation route and biomass precursor composition. Acid treatment can promote dehydration, oxidation, bond cleavage, and partial disruption of aromatic stacking, resulting in broader diffraction features and smaller coherent domains. Conversely, untreated precursors or milder activation conditions may retain partially ordered carbon domains [25,47]. However, the XRD-derived values should be interpreted cautiously: they do not represent fully crystalline nanostructures, but rather nanoscale disordered carbon domains with limited stacking coherence.

From an adsorption perspective, this structural disorder is relevant because defective carbon domains, edge sites, and irregular pore environments may enhance the apparent functional pore accessibility to DOM fractions in real lagoon water [26,48]. Thus, samples with lower apparent coherent domain sizes, such as ACPCSA5 and ACCPHSA10, are expected to provide a more disrupted and adsorption-accessible carbon framework, favoring interactions with complex oxidizable organic molecules contributing to COD [25,47].

**Figure 3:**
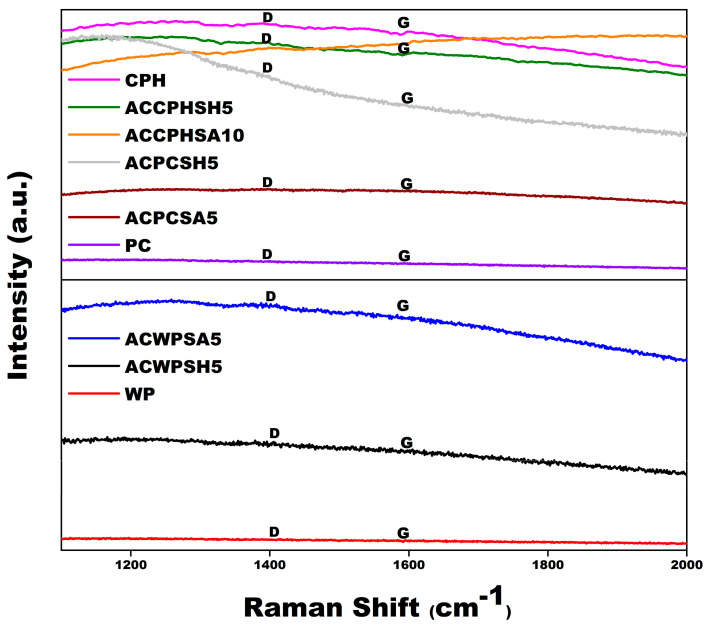
Raman spectra of Biomass-derived ACs derived from CPH, WP, and PC, showing the characteristic D and G bands associated with disordered and graphitic carbon structures, respectively.

Raman spectroscopy further supported the disordered carbonaceous nature of the materials (Figure 3). All samples exhibited the characteristic D band around ~1350 cm^−1^, associated with defects and disordered carbon domains, and the G band around ~1580 cm^−1^, assigned to in-plane vibrations of sp^2^-bonded carbon atoms [45,47].

The relative intensity ratio (I_D_/I_G_) varied among samples, ranging from 0.980 to 1.134 (Table S2). The lowest values were observed for ACCPHSA10 (0.980), ACPCSA5 (1.019), and CPH (1.029), suggesting relatively more organized sp^2^ domains. In contrast, higher I_D_/I_G_ values were obtained for ACPCSH5 (1.134), WP (1.125), and PC (1.117), indicating a higher contribution of defect-rich carbon structures [56,57,58].

Chemical treatment influenced the Raman response differently depending on the precursor. For PC-derived materials, alkaline activation increased the I_D_/I_G_ ratio relative to the raw material, suggesting enhanced defect formation and disruption of aromatic domains. In contrast, acid treatment of PC resulted in a lower I_D/_I_G_ ratio, suggesting partial stabilization or reorganization of sp^2^ domains. Similarly, acid-treated CPH (ACCPHSA10) exhibited a lower I_D_/I_G_ ratio than the raw and alkaline-treated samples. WP-derived materials showed intermediate I_D_/I_G_ values, indicating moderate structural modification regardless of activation route.

Accordingly, the Raman response was not interpreted as evidence of highly graphitized carbon, but rather as an indicator of defect-rich sp^2^ clustering, aromatic-domain reorganization, and structural disorder within the chemically activated biomass-derived carbonaceous matrix. These findings are consistent with the XRD results, which showed broad (002) reflections and nanometric apparent coherent domain sizes [59,60].

The combined XRD–Raman results indicate that the materials are neither fully graphitic nor completely amorphous. Instead, they consist of turbostratic, defect-rich carbonaceous domains whose structural organization is modulated by both precursor type and activation route [56,61]. This structural state is important for adsorption because two complementary interaction pathways may coexist: defect-rich and edge-site domains can promote hydrogen bonding, electrostatic interactions, and surface reactivity, whereas more organized aromatic sp^2^ domains may favor π–π interactions with aromatic DOM fractions, such as humic-like substances [14,16,48,57].

Therefore, adsorption performance in real lagoon water should not be attributed to structural order or disorder alone. Rather, COD removal is governed by the balance between accessible disordered domains, aromatic sp^2^ regions, and surface functional groups. This interpretation supports the central structure–property–performance relationship proposed in this study: effective DOM/COD removal depends on the integration of carbonaceous structural accessibility and surface chemistry, rather than on BET surface area alone.

Despite these insights, the interpretation of I_D_/I_G_ ratios should be approached with caution. Raman spectroscopy provides a semi-quantitative measure of disorder and does not directly quantify crystallinity, porosity, or adsorption capacity. Factors such as laser wavelength, baseline correction, and peak fitting may influence intensity ratios [52,54,62,63]. Therefore, the Raman data were interpreted together with XRD, FTIR, pH_PZC_, and adsorption results to support a multimechanistic adsorption model for complex real-water matrices.

#### 3.1.3. FTIR Characterization and Surface Functional Groups

In contrast to XRD and Raman spectroscopy, which describe the structural organization of the carbonaceous framework, FTIR was used to evaluate surface functional chemistry. This analysis focuses on oxygen-containing functional groups and aromatic surface moieties that may act as active adsorption sites for DOM. Therefore, FTIR provides the chemical basis for explaining how the structurally disordered carbonaceous domains identified by XRD and Raman can interact with DOM fractions contributing to COD.

**Figure 4:**
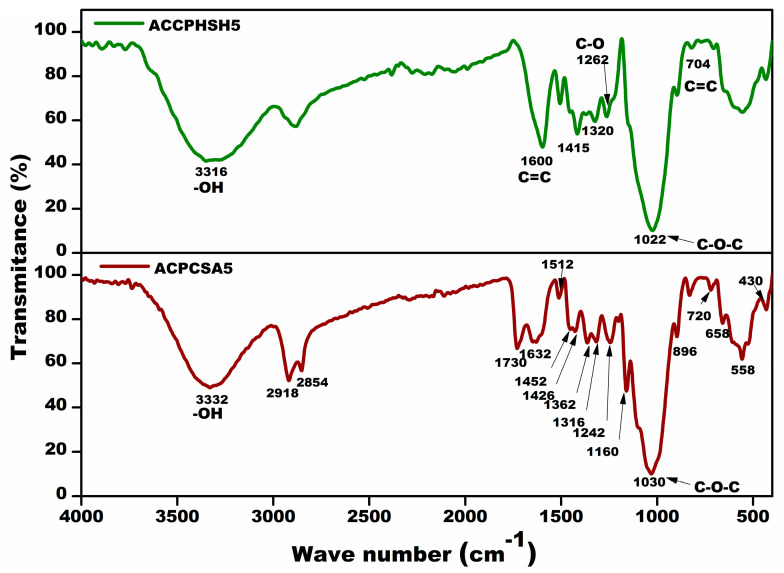
FTIR spectra of Biomass-derived ACs derived from CPH and PC (ACPCSA5 and ACCPHSH5), highlighting the surface functional groups involved in adsorption processes and their potential contribution to COD removal.

The FTIR spectra of the selected chemically treated materials, ACPCSA5 and ACCPHSH5, revealed clear differences in surface chemistry, particularly in the distribution and intensity of oxygen-containing functional groups (Figure 4). These differences are directly associated with both precursor composition and activation pathway.

Both materials exhibited a broad absorption band in the 3400–3300 cm^−1^ region, corresponding to O–H stretching vibrations associated with hydroxyl groups from cellulose, hemicellulose, phenolic structures, and adsorbed moisture. These hydroxyl functionalities provide polar surface sites that may interact with DOM through hydrogen bonding [8].

In the aliphatic region, bands near 2920–2850 cm^−1^ were assigned to C–H stretching vibrations of –CH_2_ and –CH_3_ groups originating from aliphatic chains of polysaccharides and lignin components. The moderate intensity of these bands suggests partial preservation of lignocellulosic structural fragments after chemical treatment, together with chemical modification of the original biomass matrix [64].

A band around 1730 cm^−1^, attributed to C=O stretching vibrations of carbonyl groups in carboxylic acids, esters, and other oxygenated functionalities, was more clearly defined in ACPCSA5. This result indicates a greater abundance or accessibility of carbonyl-containing groups after sulfuric acid treatment of PC. In contrast, this band was absent or strongly attenuated in ACCPHSH5, suggesting a lower contribution of carbonyl functionalities in the NaOH-treated CPH sample. These oxygenated groups are relevant because they can participate in hydrogen bonding, dipole–dipole interactions, electrostatic interactions, and surface complexation with polar organic compounds [46,65,66].

The spectral region between 1650 and 1500 cm^−1^ provided additional information on aromatic structures. In ACPCSA5, a band at approximately 1512 cm^−1^ was associated with aromatic skeletal vibrations derived from lignin-related structures. The presence of this band suggests that aromatic domains remained after chemical treatment and may contribute to π–π interactions with aromatic DOM fractions. The aromatic band around ~1500 cm^−1^ was also evident in ACCPHSH5, supporting the presence of lignin-derived aromatic domains in the CPH-based material [53,67,68].

Differences were also observed in the 1450–1300 cm^−1^ region, where bands associated with CH_2_ bending and carboxylate-related vibrations appeared. In ACPCSA5, peaks at approximately 1452 and 1426 cm^−1^ were more clearly resolved than in ACCPHSH5, suggesting a higher contribution of oxidized surface functionalities and greater surface polarity [24,41,64,69].

The most notable differences between the materials occurred in the 1200–1000 cm^−1^ region, assigned mainly to C–O and C–O–C stretching vibrations from alcohol, ether, and polysaccharide-derived groups. ACPCSA5 showed multiple bands at approximately 1160 and 1030 cm^−1^, whereas ACCPHSH5 exhibited a dominant band around 1022 cm^−1^ with fewer additional features. The richer spectral profile of ACPCSA5 in this region indicates a greater diversity of oxygen-containing functional groups and a more heterogeneous surface chemistry [28,67,70,71,72].

Additional bands below 900 cm^−1^, including peaks near 896 and 832 cm^−1^, were attributed to out-of-plane aromatic C–H vibrations and structural features of lignocellulosic polymers. These bands further support the persistence of aromatic components derived from lignin in the treated biomass [62,73,74].

Table S3 highlights the higher diversity of oxygen-containing functional groups in ACPCSA5 compared to ACCPHSH5, particularly in the carbonyl and C–O–C regions. This increased functional heterogeneity provides a broader range of interaction mechanisms with DOM, supporting the enhanced adsorption performance observed for the PC–derived material.

The FTIR spectra of the raw lignocellulosic precursors, PC, WP, and CPH, along with their corresponding chemically treated materials, are presented in Figure S1, while detailed band assignments are summarized in Table S4. All raw biomass samples showed characteristic lignocellulosic features, including broad O–H stretching bands in the 3400–3300 cm^−1^ region and aliphatic C–H stretching bands near ~2900 cm^−1^. However, precursor-dependent differences were observed. WP showed stronger signals in the carbohydrate-associated region between 1200 and 1000 cm^−1^, indicating a higher contribution of cellulose and hemicellulose fractions, whereas CPH exhibited more pronounced aromatic bands, consistent with its higher lignin contribution.

After chemical activation, changes in the intensity and distribution of oxygen-containing groups and aromatic bands confirmed the progressive modification of lignocellulosic components and the formation of carbonaceous frameworks with distinct surface functionalities. These transformations demonstrate that both precursor selection and activation route are critical for tailoring the surface chemistry of the resulting adsorbents.

Overall, ACPCSA5 exhibited the highest functional complexity, including hydroxyl, carbonyl, ether, carboxylate-related, and aromatic groups. This multifunctional surface provides multiple interaction pathways with DOM, including hydrogen bonding, dipole–dipole interactions, electrostatic contributions, and π–π interactions. While similar hydrogen-bonding and π–π interaction mechanisms have been reported in advanced adsorbent systems such as MOFs and pillararenes, including studies focused on controlled gas-phase adsorption and CO_2_/N_2_ separation, the present materials differ substantially in their design philosophy [75,76]. Rather than relying on highly ordered frameworks or molecular-recognition cavities, adsorption in the studied biomass-derived carbonaceous adsorbents appears to be governed by the synergistic contribution of apparent functional pore accessibility inferred from adsorption behavior, oxygenated surface groups, defect-rich sp^2^ domains, and favorable surface charge under real-water conditions. This comparison indicates that, whereas pillararenes and MOFs represent highly engineered adsorption platforms, the materials developed here provide a sustainable, low-cost, and matrix-relevant alternative for organic-load reduction in real eutrophic water. In contrast, ACCPHSH5 showed a less diverse surface chemistry, mainly characterized by hydroxyl and aromatic groups, with reduced carbonyl contribution and fewer C–O/C–O–C features.

The mechanistic mapping in Table S5 indicates that ACPCSA5 enables multiple interaction pathways due to its diverse functional groups, whereas ACCPHSH5 is primarily limited to hydrogen bonding and π–π interactions. This distinction is important for adsorption in real lagoon water because DOM is a heterogeneous mixture of polar compounds, such as carbohydrates, proteins, and organic acids, as well as aromatic fractions, including humic-like substances. Therefore, a surface with greater functional diversity, such as that of ACPCSA5, can interact with a broader range of DOM fractions [58,59,77,78,79]. This explains why COD removal cannot be predicted only from textural parameters, such as BET surface area, but requires consideration of adsorption-inferred functional pore accessibility, surface chemistry, structural disorder, and aromatic-domain organization.

The FTIR findings, therefore, complement the XRD, Raman, and N_2_ adsorption–desorption results. While XRD and Raman supported the presence of turbostratic, defect-rich carbonaceous domains, FTIR demonstrated that these domains are decorated with oxygen-containing and aromatic functional groups that can act as adsorption-active sites. In parallel, N_2_ adsorption–desorption analysis provided textural evidence of narrow pore domains and pore size distribution, although the low BET surface areas indicate that total surface area alone is not sufficient to explain COD removal. Together, these results support the proposed multimechanistic adsorption model, in which COD removal from real eutrophic lagoon water is governed by the combined contribution of apparent functional pore accessibility inferred from adsorption behavior, oxygenated surface functionalities, aromatic sp^2^ regions, defect-rich carbonaceous domains, pore size distribution, and surface charge behavior.

#### 3.1.4. UV–Vis Diffuse Reflectance Analysis

**Figure 5:**
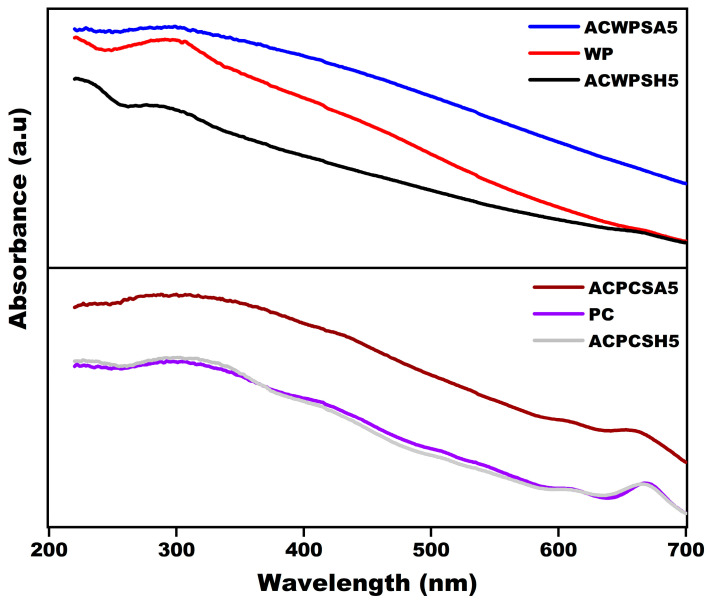
UV–Vis DR spectra of the raw lignocellulosic precursors, PC, and WP, and Biomass-derived ACs from CPH, and WP, illustrating their optical absorption behavior and electronic transitions associated with carbonaceous materials.

UV evaluated the optical properties of the synthesized carbon materials—Vis Diffuse Reflectance spectroscopy in the 200–700 nm range (Figure 5). All samples exhibited a broad absorption profile, characterized by strong absorption in the ultraviolet region followed by a gradual decrease toward the visible range. This behavior is typical of carbonaceous materials with disordered or turbostratic structures, where a continuous distribution of electronic states replaces a well-defined bandgap [47,56,60].

In the WP series (Figure 5), clear differences in absorbance intensity were observed among the samples. ACWPSA5 displayed the highest absorbance across the entire spectral range, followed by WP and ACWPSH5, with the most pronounced differences in the UV region (200–350 nm). Similarly, in the PC series (Figure 5), ACPCSA5 exhibited the highest absorbance throughout the spectrum, particularly below 400 nm, while PC and ACPCSH5 showed lower and comparable profiles with only minor deviations at longer wavelengths. Additionally, subtle spectral features were identified in the 250–320 nm region and weak shoulders around 600–700 nm, suggesting the presence of heterogeneous electronic transitions associated with oxygenated functional groups and structural disorder [80].

These experimental results demonstrate that both the precursor type and the chemical activation route strongly influence the optical response of the materials, as reflected in variations in both absorbance intensity and spectral shape. In particular, the consistently higher absorbance observed for acid-treated samples (ACWPSA5 and ACPCSA5) indicates a significant modification of their electronic structure.

From a mechanistic perspective, the enhanced absorption can be attributed to an increased density of delocalized π-electrons associated with the development of aromatic and graphitic domains during chemical activation. Acid treatments are known to promote dehydration, condensation, and aromatization reactions, leading to the formation of conjugated carbon networks and oxygenated surface groups. These structural transformations directly impact the electronic transitions observed in UV–Vis spectra [48,81].

The strong absorption in the UV region (200–350 nm) is primarily associated with π → π* transitions of aromatic C=C bonds, while contributions from n → π* transitions related to oxygen-containing functional groups (e.g., C=O, –COOH) are also expected [59,82]. The higher absorbance of ACPCSA5 compared to PC and ACPCSH5 suggests that acid activation enhances both conjugation and surface functionality, resulting in more efficient electronic transitions [52].

Moreover, the gradual decay of absorbance toward the visible region, without a sharp absorption edge, confirms the absence of a well-defined bandgap. This behavior is characteristic of amorphous or turbostratic carbons, where localized and delocalized electronic states coexist due to structural disorder. Such electronic features are consistent with biomass-derived ACs, whose properties depend strongly on synthesis conditions and precursor composition [25,47].

Importantly, these optical properties have direct implications for adsorption performance in real water matrices. Carbon materials with enhanced π-electron systems and surface functionalities exhibit stronger interactions with DOM, particularly aromatic fractions detectable by UV_254_. These interactions are dominated by π–π stacking, hydrophobic interactions, and electrostatic effects, which are widely recognized as key mechanisms in pollutant removal by carbon-based adsorbents [8,32,83].

An additional relevant observation is the similar absorbance behavior between PC and ACPCSH5, suggesting that alkaline activation does not significantly enhance the electronic structure beyond that of the raw precursor. This may indicate a threshold effect, where excessive treatment does not linearly improve conjugation and may even induce competing phenomena such as partial structural collapse or disruption of aromatic domains.

Overall, the UV–Vis DR analysis provides strong evidence that acid activation (particularly in ACPCSA5) leads to the most pronounced electronic delocalization, which is consistent with its superior adsorption performance observed experimentally. This reinforces the structure–property–performance relationship linking chemical activation → electronic structure → DOM/COD removal efficiency, a key aspect for understanding and optimizing biomass-derived ACs for real wastewater treatment.

To quantitatively assess these electronic changes, the reflectance data were transformed using the Kubelka–Munk function, F(R), and Tauc-type plots were constructed to estimate apparent optical transition energies. The optical parameters estimated from diffuse reflectance data were interpreted cautiously due to the heterogeneous and disordered nature of the biomass-derived carbonaceous materials. Therefore, the Tauc-derived values are reported as apparent optical band gaps rather than definitive semiconductor band gaps. These values provide complementary information on changes in optical absorption associated with chemical activation, carbonization degree, and electronic disorder, but they are not used as primary descriptors of adsorption performance [47,80]. The adsorption behavior discussed in this work is instead interpreted mainly from the combined evidence derived from FTIR, pH_PZC_, Raman spectroscopy, XRD, UV–Vis DR, BET/pore-size analysis, and COD removal in real lagoon water.

The estimated bandgap values ranged from 2.41 to 2.15 eV, revealing a clear dependence on both precursor type and activation chemistry.

The electronic trends summarized in Table S6 are consistent with well-established relationships between chemical activation, carbon structure, and optical properties in biomass-derived carbons. The observed decrease in apparent bandgap following chemical treatment, particularly under acidic conditions, is widely attributed to dehydration and aromatization processes that promote the formation of extended π-conjugated domains and sp^2^-hybridized carbon networks. These structural transformations increase electronic delocalization and generate defect-related energy states within the carbon matrix, resulting in enhanced light absorption and reduced optical bandgap values. Similar behavior has been reported in recent studies, where acid-AC exhibited higher aromaticity and improved electronic conductivity compared to their raw or alkali-treated counterparts [8,67,84]. In contrast, alkaline activation primarily enhances porosity and surface functionalization, but induces a less pronounced reorganization of the electronic structure, which explains the intermediate bandgap values observed for NaOH-treated samples. Therefore, the progressive decrease in bandgap from raw to acid-treated materials reflects a transition from insulating lignocellulosic matrices toward more conjugated and electronically active carbon frameworks, which are known to favor π–π interactions with aromatic DOM and enhance adsorption performance in aqueous systems [47,85].

The observed bandgap trend, ACPCSA5 < WPSA5 < ACCPHSH5 < raw biomasses, reflects differences in structural ordering and π-electron delocalization. Lower bandgap values suggest higher aromaticity, a greater contribution of sp^2^-hybridized carbon domains, increased electronic mobility, and stronger π–π interaction capability [47]. These features are particularly relevant for the adsorption of aromatic DOM fractions, commonly associated with UV_254_, and also contribute to the reduction of the broader organic load measured as COD [63,86]. Accordingly, ACPCSA5, which exhibited the lowest bandgap and the highest degree of electronic delocalization, is expected to interact more effectively with aromatic and recalcitrant DOM components through π–π stacking and hydrophobic interactions. In contrast, raw or less conjugated materials with higher bandgap values are expected to show weaker π–π interactions and lower adsorption efficiency. Therefore, the electronic structure of the carbonaceous adsorbents helps explain the superior COD removal observed for ACPCSA5, while recognizing that COD includes both aromatic and non-aromatic organic fractions.

#### 3.1.5. Point of Zero Charge (pH_PZC_) Determination

**Figure 6:**
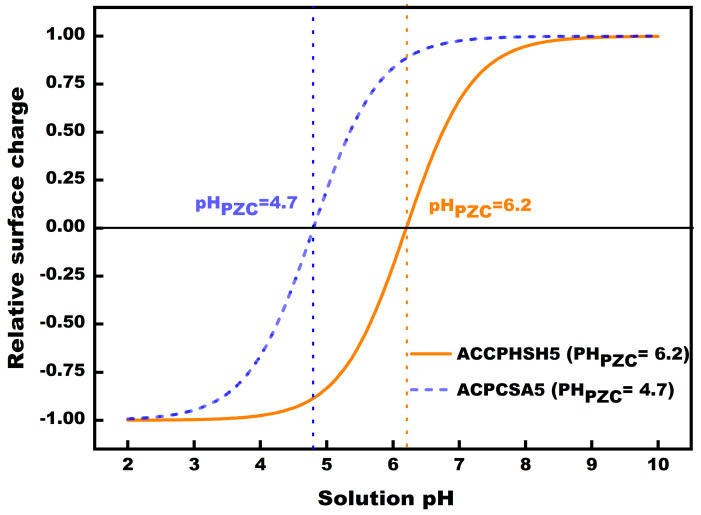
Determination of the pH_PZC_ of ACPCSA5 and ACCPHSH5 using the pH drift method.

The pH at the pH_PZC_ provides important insight into the surface charge behavior of the adsorbents and their effect on removing organic matter from the lagoon matrix. This parameter is particularly relevant because the adsorption assays with real lagoon water were conducted at pH 6. When the solution pH is below the pH_PZC_, the adsorbent surface is predominantly positively charged, whereas at pH values above the pH_PZC_ the surface becomes negatively charged [19,26]. The points where ΔpH = 0 indicate the pH_PZC_. As shown in Figure 6, ACPCSA5 has a lower pH_PZC_ (~4.7), while ACCPHSH5 has a value closer to neutrality (~6.2), reflecting the different surface chemistries caused by acid and alkaline treatments, respectively. Under the lagoon water conditions (pH ≈ 6), ACPCSA5 is likely to have a mostly negatively charged surface. Meanwhile, ACCPHSH5 remains near its zero-charge point, with a nearly neutral surface or slightly positive under the operating conditions. This suggests that electrostatic interactions alone cannot fully explain the observed COD removal trends. Instead, adsorption probably results from a mix of mechanisms, including pore filling within the hierarchical carbon structure, π–π interactions between aromatic parts of DOM and graphitic carbon surfaces, hydrogen bonding with oxygen-containing functional groups, and hydrophobic interactions [19,26,49,50]. This fits with the heterogeneous nature of DOM, which includes a mixture of neutral, anionic, and hydrophobic fractions that interact differently with the adsorbent surface [3,68,77]. Therefore, the closeness of the operating pH to the pH_PZC_, especially for ACCPHSH5, emphasizes the importance of non-electrostatic interactions in controlling adsorption performance, reinforcing the importance of surface chemistry and porosity in removing complex organic matter from real water matrices. Additionally, the high efficiency seen in COD reduction can be linked to the ability of AC materials to adsorb multiple fractions of organic matter at once, as widely reported for lignocellulosic-derived carbons [3,24,25].

Only a minor fraction of DOM in natural waters is positively charged at circumneutral pH. This fraction is typically associated with protonated amine-containing compounds, such as peptides and microbial-derived organic matter, and generally represents less than 5% of the total DOM pool. In contrast, the dominant components of DOM, including humic and fulvic substances, exhibit predominantly negative charge due to the presence of deprotonated carboxylic and phenolic groups [25,68,77,78]. Therefore, despite ACPCSA5 exhibiting a negatively charged surface at the operating pH (pH ≈ 6), electrostatic attraction toward positively charged species is expected to contribute only marginally to the overall adsorption process. The high COD removal observed must instead be attributed to non-electrostatic mechanisms, including π–π interactions with aromatic moieties, pore-filling within the carbon structure, hydrogen bonding, and hydrophobic interactions, which are known to govern the adsorption of heterogeneous organic matter in real water matrices [12,78].

### 3.2. Organic Load and Adsorption Performance in the Real Lagoon Matrix

The initial organic load of the real lagoon matrix, quantified as COD_0_, showed marked temporal variability during the sampling period. COD_0_ values ranged from relatively low organic-load conditions during the rainy season (<100 mg L^−1^) to highly polluted conditions during the dry season, reaching 2000–5000 mg L^−1^. This variability is consistent with the hydrological behavior of urban lagoons, where dilution during rainy periods and organic matter accumulation during low-water-renewal conditions strongly affect water quality. The highest COD_0_ values indicate episodic contamination events, likely associated with urban runoff, untreated wastewater inputs, and localized accumulation of organic matter in poorly mixed zones.

Because the study was based on discrete sampling campaigns rather than continuous monitoring, the dataset should be interpreted as representative of contrasting real-matrix conditions rather than as a full seasonal model. Hydrological variables such as precipitation intensity, residence time, and flow exchange were not directly measured. Therefore, the adsorption results are discussed in relation to the experimentally measured COD_0_ values, with performance comparisons restricted to materials evaluated under comparable initial organic-load conditions.

#### 3.2.1. COD Removal Performance and Apparent Adsorption Kinetics

COD removal from real lagoon water was evaluated using raw and chemically activated carbonaceous adsorbents derived from CPH, WP, and PC. All batch experiments were conducted under comparable treatment conditions within each experimental series: pH ≈ 6, adsorbent dosage of 4 g L^−1^, and contact time up to 180 min (Table 2). Because the initial COD varied between sampling campaigns, the adsorption results were grouped according to COD_0_ to avoid overinterpretation of concentration-dependent removal efficiencies.

For the COD_0_ = 2000 mg L^−1^ series, raw CPH achieved 60.0 ± 1.3% COD removal, whereas raw WP showed a much lower removal efficiency of 16.0 ± 2.1%. After chemical activation, the adsorption performance increased markedly. The alkaline-treated ACCPHSH5 and ACWPSH5 reached 80.7 ± 1.2% removal, while the acid-treated ACWPSA5 achieved the highest efficiency within this COD_0_ range, with 89.7 ± 1.2%. Therefore, the performance trend within the COD_0_ = 2000 mg L^−1^ series was:

ACWPSA5 > ACWPSH5 ≈ ACCPHSH5 > raw CPH > raw WP.

For the COD_0_ = 5000 mg L^−1^ series, raw PC achieved 58.0 ± 1.0% COD removal, whereas the acid-activated ACPCSA5 exhibited near-complete COD removal, reaching 98.7 ± 1.2% after 180 min. This result confirms that H_2_SO_4_ activation substantially improved the adsorption performance of the pineapple-derived material under high organic-load conditions. However, this value is not directly ranked against the materials tested at COD_0_ = 2000 mg L^−1^; instead, it is discussed as the best-performing material within the COD_0_ = 5000 mg L^−1^ experimental series.

**Figure 7:**
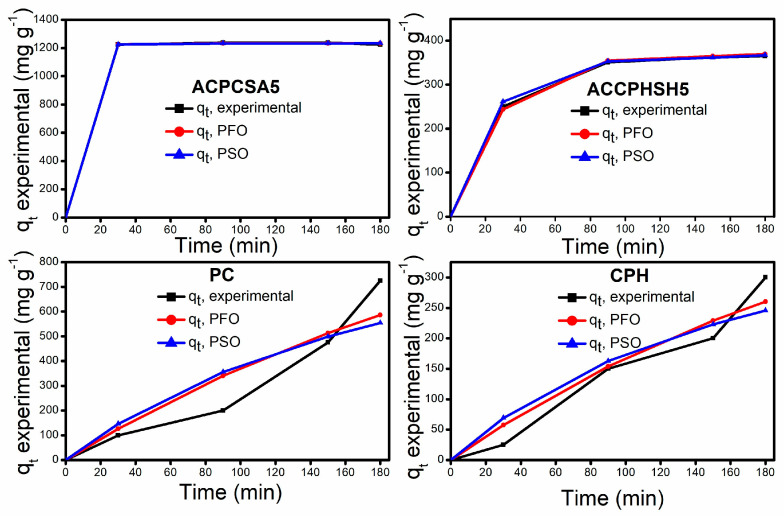
Nonlinear fitting of COD adsorption kinetics for ACPCSA5, ACCPHSH5, PC, and CPH in real lagoon water using PFO and PSO models. Experimental *q*ₜ values are shown for comparison.

The nonlinear kinetic fitting further supported the strong effect of chemical treatment on COD removal behavior (Figure 7; Table S7). Because COD represents a collective water-quality parameter associated with a heterogeneous mixture of dissolved, colloidal, and possibly particulate organic species, the PFO and PSO models were used only as empirical descriptors of apparent removal trends. They should not be interpreted as definitive evidence of a single adsorption mechanism.

ACPCSA5 exhibited the fastest apparent COD uptake. Most of the COD-contributing fraction accessible to this material was removed within the first 30 min, after which the adsorption capacity remained nearly constant up to 180 min. This rapid plateau-like behavior suggests highly accessible interaction sites and efficient mass transfer within the real lagoon matrix. ACCPHSH5 also showed rapid initial uptake, followed by a slower increase toward apparent equilibrium, indicating a combination of fast surface interactions and accessibility-controlled removal processes. In contrast, raw PC and CPH showed slower, progressive COD uptake and did not reach a clear equilibrium plateau within the experimental window.

Overall, the COD-removal and kinetic trends demonstrate that chemical activation improved both removal efficiency and apparent uptake rate. The results also indicate that adsorption behavior in real lagoon water was governed by the interaction between biomass precursor and activation chemistry, rather than by the raw biomass composition alone.

#### 3.2.2. Comparison with Literature and Practical Relevance

The adsorption performance observed in this study is consistent with previous reports showing that chemical activation can strongly improve the removal capacity of lignocellulosic waste-derived adsorbents. Alkali-pretreated sugarcane bagasse, corn husk, and rice husk have been reported to achieve high methylene blue removal efficiencies, while sulfuric-acid-activated watermelon rind and magnetic watermelon-shell ACs have shown high adsorption capacities toward model dyes. Similar improvements have also been reported for ACs derived from banana stem, coffee husk, and cocoa pod husk, where activation enhanced pore development, surface functionality, and pollutant uptake [19,23,31,67]. To further contextualize the performance of the developed carbons, Table S8 summarizes representative adsorption results reported for Biomass-derived ACs, with particular emphasis on studies conducted in real or complex aqueous matrices.

However, direct comparison with these studies must be made cautiously. Most literature reports evaluate adsorption using single-solute synthetic systems, commonly dyes such as methylene blue or methyl orange, under controlled laboratory conditions. In contrast, the present work evaluates COD removal from a real eutrophic urban lagoon matrix. COD does not represent a single adsorbate, but rather a global parameter associated with heterogeneous oxidizable organic matter, including dissolved and colloidal fractions. Therefore, the near-complete COD removal achieved by ACPCSA5 is particularly relevant because it was obtained under chemically complex conditions where DOM heterogeneity, inorganic ions, suspended species, and competitive adsorption effects may limit performance.

The superior behavior of ACPCSA5 can be reasonably attributed to a synergistic combination of (i) BJH-indicating narrow pore architecture and apparent functional pore accessibility inferred from adsorption behavior, (ii) a higher density of oxygen-containing surface functionalities, and (iii) more adsorption-accessible aromatic domains capable of interacting with complex dissolved organic fractions in lagoon water. This behavior agrees with the broader literature indicating that oxidized or functionalized carbon surfaces improve the adsorption of organic pollutants through multiple concurrent mechanisms, including hydrogen bonding, donor–acceptor interactions, electrostatic contributions, and π–π stacking.

Within this context, the main contribution of the present study is not only the high removal efficiency, but also the demonstration that waste-derived chemically activated carbonaceous adsorbents can remain effective in a real aqueous matrix. ACPCSA5 stood out as the most promising material, since the acid-activated PC adsorbent achieved COD removal comparable to, or higher than, values commonly reported for biomass-derived adsorbents under idealized single-solute conditions, even though it was evaluated in a more complex real lagoon matrix. Therefore, its performance under high organic load supports its potential application for tertiary polishing or emergency treatment during episodic contamination events in eutrophic urban lagoons.

#### 3.2.3. COD-Derived Microbial Regrowth Potential Index (MRPI)

Although bacteriological indicators were not directly measured, the strong decrease in COD after adsorption indicates a reduction in bulk oxidizable organic matter, which may decrease the pool of substrates potentially available for heterotrophic microbial regrowth. In this study, the MRPI was used only as a COD-derived proxy of residual organic-load availability. Accordingly, MRPI should not be interpreted as direct evidence of microbial inactivation, biological stabilization, or absence of biodegradable organic fractions. COD does not specifically quantify AOC, BDOC, or low-molecular-weight labile compounds. Therefore, the MRPI values reported here are conservative indicators of substrate reduction, not validated biological regrowth measurements.

Under the evaluated conditions, the chemically activated adsorbents substantially reduced MRPI. ACCPHSH5 decreased COD from approximately 2000 mg L^−1^ to 100–600 mg L^−1^, while ACPCSA5 reduced COD from approximately 5000 mg L^−1^ to values near or below the analytical detection limit. These results correspond to MRPI values ranging from approximately 0.25 for ACCPHSH5 to near 0.00 for ACPCSA5, equivalent to an estimated reduction in residual oxidizable substrate availability of approximately 75% to ≥98%. This strong reduction was especially evident for ACPCSA5, although direct biological validation remains necessary.

Future work should therefore include 48–72 h regrowth assays, heterotrophic plate counts, ATP-based activity measurements, flow cytometry, AOC/BDOC analysis, TOC/DOC fractionation, molecular-weight fractionation, and UV254 monitoring. These analyses are necessary to determine whether COD reduction translates into lower biodegradable organic carbon availability and reduced biological regrowth under real environmental conditions.

### 3.3. Integrated Structure–Property Performance Relationship Governing DOM Adsorption and COD Removal

The adsorption performance of the Biomass-derived chemically activated carbonaceous adsorbents was governed by the combined effect of apparent functional pore accessibility, surface chemistry, structural disorder, electronic properties, and surface charge, rather than by BET surface area alone (Figure S2). Although all materials exhibited low N_2_-BET surface areas, their COD removal efficiencies differed markedly, confirming that total surface area was not an adequate standalone predictor of adsorption behavior in the real lagoon matrix.

The superior performance of ACPCSA5 can be rationalized by integrating the XRD, Raman, FTIR, UV–Vis DRS, pHPZC, and adsorption results. The markedly higher COD removal achieved by ACPCSA5, despite its low BET surface area, suggests greater apparent functional accessibility of adsorption domains under real aqueous conditions. This apparent accessibility is inferred from adsorption behavior and mass-transfer performance rather than from direct morphological observations. XRD and Raman analyses indicated disordered turbostratic carbon domains and partially developed sp^2^-hybridized regions, which may contribute to π-related interactions with aromatic and humic-like organic matter. The lower optical band gap of ACPCSA5 further suggests enhanced π-electron delocalization, supporting stronger interactions with conjugated organic structures.

In parallel, FTIR confirmed the presence of oxygen-containing functional groups, including hydroxyl, carbonyl, and ether-type functionalities, which can promote hydrogen bonding, dipole–dipole interactions, and polar DOM adsorption, while the disordered sp^2^-rich carbon domains may promote π–π interactions with aromatic DOM fractions present in the lagoon water.

Surface charge also contributed to the adsorption response. The pHPZC of ACPCSA5 lies below the lagoon-water pH, indicating that the material surface was predominantly negatively charged under the experimental conditions. This condition may favor electrostatic interactions with protonated or positively charged organic species, while oxygenated functionalities and aromatic domains simultaneously support polar and π-related interactions. Therefore, COD removal in this real matrix should be interpreted as a multimechanistic process involving apparent functional accessibility of adsorption domains, oxygen-containing surface groups, disordered aromatic carbon regions, and surface-charge effects.

Importantly, the chemically treated materials are not discussed here as conventional high-surface-area activated carbons. Rather, they are better described as chemically activated biomass-derived carbonaceous adsorbents exhibiting multifunctional surface chemistry, disordered aromatic domains, and adsorption behavior indicative of accessible adsorption sites under real aqueous conditions. Although sub-2 nm pore features and the 1.16–2.59 nm apparent coherent domain sizes estimated from XRD provide indirect evidence of nanoscale structural organization, the term “nanostructured” is used cautiously because TEM or HRTEM analyses were not performed.

The integrated characterization and adsorption results indicate that ACPCSA5 provided the most favorable balance of apparent functional pore accessibility, surface functionality, disordered sp^2^-rich domains, electronic delocalization, and surface charge. This combination explains its rapid and near-complete COD removal under high-organic-load lagoon water conditions. In contrast, raw biomasses showed lower and slower removal, while other chemically activated materials achieved intermediate performance depending on precursor type and activation route.

These findings demonstrate that adsorption efficiency in complex real-water systems is controlled by an optimized combination of structural and chemical characteristics rather than by maximum surface area, graphitization degree, or structural disorder alone. Overall, the results support the rational design of biomass-derived carbonaceous adsorbents from tropical agro-industrial residues for realistic water treatment applications requiring multifunctional adsorption mechanisms rather than purely high-surface-area materials.

### 3.4. Preliminary Economic Assessment

A comparison of economic metrics reported in biomass-derived AC studies reveals a persistent limitation in the field: the lack of standardized cost normalization, particularly in terms of USD kg^−1^ of adsorbent. Most studies qualitatively classify their materials as “low-cost” or “cost-effective” without providing quantitative economic indicators that allow direct comparison across systems [18,24,27,31,53,87]. This gap hinders the translation of laboratory-scale developments into techno-economically comparable solutions for real water treatment applications.

An exception is the study by Benjelloun et al. [51], who reported a process-level treatment cost of approximately 1.0484 USD L^−1^ for dye removal using AC derived from *Capparis spinosa* waste. Their analysis demonstrated that chemical reagents and thermal energy dominate the overall cost structure, whereas the biomass precursor contributes only marginally. This finding is consistent with the broader literature on lignocellulosic valorization, in which feedstock abundance and low acquisition cost are key advantages of biomass-based adsorbents [25].

In line with these observations, the present study adopts a normalized cost framework and proposes a medium-case production estimate of 2.15 USD kg^−1^ for the synthesized structured AC. This value was derived from a techno-economic breakdown in which chemical activation, washing/neutralization steps, and energy consumption represent the primary cost drivers, while the biomass feedstock accounts for only a minor fraction of total cost. Such distribution reinforces the notion that economic optimization strategies should primarily target process intensification and reagent/energy efficiency rather than raw material substitution.

Furthermore, recent advances in biomass-derived ACs, including CPH-based materials, support the feasibility of producing high-performance adsorbents from lignocellulosic residues within a circular economy framework, even when explicit USD kg^−1^ values are not consistently reported in the literature. This highlights the importance of integrating performance metrics (e.g., COD removal efficiency) with normalized economic indicators, enabling more robust comparisons with commercial AC and facilitating scale-up assessments.

Assuming a single-use operation without regeneration and an adsorbent dose of 4 g L^−1^ (4 kg m^−3^), the estimated treatment cost was USD 2.0–2.8 m^−3^ under a low-cost precursor scenario and USD 8.6 m^−3^ under a medium-cost scenario. The low-cost scenario represents an optimistic estimate based mainly on bulk adsorbent value, whereas the medium-cost scenario more realistically reflects chemical activation with NaOH or H_2_SO_4_, including washing/neutralization, water use, and energy demand. Under the same dosage, commercial AC priced at USD 5–15 kg^−1^ would yield a treatment cost of USD 20–60 m^−3^, indicating a clear economic advantage for waste-derived ACs. This estimation aims to provide an initial insight into the feasibility of implementing waste-derived ACs for real-water treatment applications [30].

On a performance basis, the cost of COD abatement was estimated at USD 1.25–1.75 per kg COD removed ×10 ^−3^ for a representative case of 2000 mg L^−1^ initial COD with 80% removal, and USD 0.41–0.57 per kg COD removed for a high-load event of 5000 mg L^−1^ with 98% removal; the corresponding value for the medium-cost scenario was USD 5.38 and USD 1.76 per kg COD removed ×10 ^−3^, respectively (Table 3). Thus, the apparent cost efficiency improves under high organic-load conditions, provided that high COD removal is maintained. This behavior is particularly relevant for episodic contamination events in urban lagoons, where influent organic loads may increase substantially [14].

These results are consistent with previous studies indicating that the main economic limitations of AC production arise not from biomass cost, but from chemical activation, washing, and energy consumption steps [30,88].

These scenarios allow benchmarking against commercial AC and evaluating the economic competitiveness of Biomass-derived ACs (Table 4).

Assuming a modular treatment system processing 10 m^3^/day for 22 days/month, the projected annual treatment capacity is 2640 m^3^/year. Under these conditions, the system could remove between 4.2 and 12.9 tonnes of COD per year, depending on the influent organic load.

The corresponding annual treatment cost ranges from:

- USD 5280–7392 year^−1^ (low-cost precursor scenario).
- USD 22,704 year^−1^ (medium-cost precursor scenario).

In comparison, using commercial AC would increase annual costs to approximately USD 52,800–158,400 year^−1^, further emphasizing the economic advantage of Biomass-derived ACs.

Overall, this preliminary techno-economic assessment indicates that the materials developed in this study should be interpreted as a low-cost, precursor-based adsorbent platform rather than a fully validated low-cost treatment process. The estimated material-use cost of 8.6 USD m^−3^ under the medium-cost scenario reflects the conservative assumption of single-use operation at 4 g L^−1^. When normalized by COD removal, the cost decreases from 5.38 USD kg^−1^ COD under moderate-load conditions to 1.76 USD kg^−1^ COD under high-load conditions, highlighting the importance of influent organic load and removal efficiency in determining economic feasibility. However, future work must evaluate adsorbent recovery, regeneration, reuse cycles, column operation, and life-cycle costs before the process can be considered economically competitive at full scale.

## 4. Conclusions

This study demonstrated that biomass-derived chemically activated carbonaceous adsorbents prepared from CPH, WP, and PC can effectively reduce the COD of real eutrophic lagoon water. The results show that adsorption performance in this complex aqueous matrix was not governed by N_2_-BET surface area alone but by an integrated balance among BET/BJH-derived textural features, pore size distribution, apparent functional pore accessibility inferred from adsorption behavior, surface chemistry, structural disorder, electronic delocalization, and surface charge.

Among the evaluated materials, the acid-activated PC adsorbent, ACPCSA5, exhibited the most promising performance, achieving near-complete COD removal under real high-organic-load lagoon-water conditions. This response was associated with its narrow pore-size features indicated by N_2_ adsorption–desorption analysis, oxygen-containing surface functionalities, partially developed sp^2^ carbon domains, lower optical band gap, and favorable surface charge at lagoon-water pH. Together, these features enabled multimechanistic interactions with heterogeneous DOM, including hydrogen bonding, dipole–dipole interactions, electrostatic contributions, hydrophobic affinity, and π–π interactions with aromatic DOM fractions.

Importantly, the materials investigated here should not be interpreted as conventional high-surface-area ACs. Instead, they are more accurately described as waste-derived chemically activated carbonaceous adsorbents with adsorption-accessible narrow pore domains, disordered aromatic carbon regions, and multifunctional surface chemistry. Although sub-2 nm pore-size values derived from N_2_ adsorption–desorption analysis and XRD-derived coherent domain sizes provide indirect evidence of narrow pore features and nanoscale disordered carbon domains, the term “nanostructured” should be used cautiously in the absence of direct TEM or HRTEM confirmation.

The MRPI analysis was introduced only as a conservative substrate-based proxy derived from the residual COD fraction. Therefore, MRPI should not be interpreted as a direct microbiological measurement or as a validated biological indicator of microbial regrowth. Nevertheless, the strong COD reduction achieved by ACPCSA5 suggests a meaningful decrease in the pool of oxidizable organic substrates available in the treated lagoon water, supporting its potential relevance for tertiary polishing or emergency remediation of eutrophic urban water bodies.

A preliminary techno-economic assessment indicated a material-use cost of 8.6 USD m^−3^ under a conservative single-use scenario at an adsorbent dose of 4 g L^−1^. This corresponded to 5.38 and 1.76 USD kg^−1^ COD removed under moderate- and high-load conditions, respectively, suggesting that the material may be more attractive for high-organic-load episodes where larger COD reductions are achieved per unit mass of adsorbent.

Overall, this work provides a realistic evaluation framework for biomass-derived carbonaceous adsorbents beyond model-pollutant systems. The findings support the rational design of low-cost, waste-derived adsorbents for complex real-water matrices, where DOM heterogeneity, competitive adsorption, apparent functional pore accessibility, pore size distribution, and surface chemistry collectively determine treatment performance. Future studies should include direct microbial regrowth assays, TOC and UV_254_ monitoring, fluorescence excitation–emission matrix spectroscopy, molecular-level DOM characterization, leaching tests, regeneration and reuse cycles, particle-release assessment, and continuous-flow column experiments to validate the long-term stability, environmental safety, scalability, and practical applicability of these materials.

## Figures and Tables

**Table 1:** Textural properties of raw biomass (CPH, WP, PC) and derived ACs.

Adsorbent	SBET (m^2^ g^−1^)	Micropore Volume (cm^3^ g^−1^)	Total/Small Pore Volume (cm^3^ g^−1^)	Average Pore Size (nm)
CPH	1.1260	0.000343	N.D.	0.413
WP	0.5807	0.000141	N.D.	0.421
PC	0.7437	0.000089	N.D.	0.442
ACWPSA5	0.5894	0.000424	N.D.	0.377
ACPCSA5	0.8026	0.000090	0.000019	1.720
ACCPHSH5	0.8299	0.001692	0.000654	3.150/0.315 ^1^

^1^ Pore size is method-dependent: 3.15 nm (BET) and 0.315 nm (MP), indicating coexisting micro and mesoporous structures.

**Table 2:** Comparative COD removal performance of raw and chemically activated lignocellulosic-derived carbonaceous adsorbents obtained from CPH, PC, and WP under batch conditions. Experimental conditions: pH ≈ 6, contact time = 180 min, adsorbent dosage = 4 g L^−1^, matrix type = real water from Laguna de las Ilusiones, Villahermosa, Tabasco, Mexico. Initial COD concentrations (COD_0_) were grouped by experimental series to avoid direct comparison between different organic-load ranges. Removal values are reported as mean ± standard deviation (*n* = 3).

COD_0_ Range ^1^	Material	Treatment	COD_0_ (mg L^−1^)	COD_180_ (mg L^−1^)	Removal (%)
2000 mg L^−1^ series	WP	Raw	2000	1690	16.0 ± 2.1
2000 mg L^−1^ series	CPH	Raw	2000	800	60.0 ± 1.3
2000 mg L^−1^ series	ACCPHSH5	NaOH 5 M	2000	600–100	80.7 ± 1.2
2000 mg L^−1^ series	ACWPSH5	NaOH 5 M	2000	360–400	80.7 ± 1.2
2000 mg L^−1^ series	ACWPSA5	H_2_SO_4_ 5 M	2000	190–230	89.7 ± 1.2
5000 mg L^−1^ series	PC	Raw	5000	2100	58.0 ± 1.0
5000 mg L^−1^ series	ACPCSA5	H_2_SO_4_ 5 M	5000	0–100	98.7 ± 1.2

^1^ COD removal efficiencies are concentration-dependent; therefore, materials evaluated at COD_0_ = 2000 mg L^−1^ and COD_0_ = 5000 mg L^−1^ are not directly ranked against each other. Quantitative comparisons are restricted to materials tested within the same COD_0_ range.

**Table 3:** Preliminary single-use treatment cost and COD abatement cost under normalized assumptions.

Scenario	Adsorbent Cost (USD kg^−1^)	Adsorbent Dose (kg m^−3^)	Treatment Cost (USD m^−3^)	Cost at 2000 mg L^−1^ COD, 80% Removal (USD kg^−1^ COD)	Cost at 5000 mg L^−1^ COD, 98% Removal (USD kg^−1^ COD)
Biomass-derived adsorbent, low-cost precursor scenario	0.50–0.70	4.00	2.00–2.80	1.25–1.75	0.41–0.57
Biomass-derived adsorbent, medium-cost precursor scenario	2.15	4.00	8.60	5.38	1.76
Commercial AC benchmark, single-use assumption ^1^	5.00–15.00	4.00	20.00–60.00	12.50–37.50	4.08–12.24

^1^ The commercial AC benchmark was normalized to the same dose used in this study. It does not include regeneration credits. In practical granular activated carbon systems, multiple regeneration cycles may reduce the effective adsorbent cost per cycle. Because regeneration was not experimentally evaluated for the waste-derived adsorbents in this work, all values are reported as conservative single-use estimates.

**Table 4:** Estimated annual COD removal and material-use cost for a modular 10 m^3^ day^−1^ treatment scenario.

Parameter	Case A	Case B
Influent COD (mg L^−1^)	2000	5000
COD removal (%)	80	98
COD removed (kg m^−3^)	1.60	4.90
Annual treated volume (m^3^ year^−1^)	2640	2640
Annual COD removed (kg year^−1^)	4224	12,936
Annual COD removed (tonnes year^−1^)	4.22	12.94
Annual material-use cost, low-cost precursor scenario (USD year^−1^)	5280–7392	5280–7392
Annual material-use cost, medium-cost scenario (USD year^−1^)	22,704	22,704
Annual material-use cost, commercial AC single-use benchmark (USD year^−1^)	52,800–158,400	52,800–158,400

## Data Availability

The original contributions presented in this study are included in the article/Supplementary Material. Further inquiries can be directed to the corresponding authors.

## Supplementary Materials

The following supporting information can be downloaded at: https://www.mdpi.com/article/10.3390/ma19132743/s1, Section S1: Adsorption Kinetics; Section S2: Experimental Basis and Boundary Conditions; Figure S1: FTIR spectra of the raw lignocellulosic precursors, PC, WP, and CPH, and waste-derived AC derived from CPH and PC, highlighting the surface functional groups involved in adsorption processes and their potential contribution to COD removal; Figure S2: Conceptual schematic of the proposed adsorption mechanism based on the experimental results discussed in this study. The figure provides an illustrative summary of the structure–property–performance relationships associated with pore accessibility, surface oxygen-containing functional groups, carbon structural disorder, surface charge, and COD removal performance. It does not contain experimental micrographs, raw characterization data, or AI-generated scientific results, and should not be interpreted as direct experimental imaging; Table S1: XRD-derived structural parameters of biomass-derived carbons estimated using the Scherrer equation; Table S2 Raman spectral parameters and structural disorder (I_D_/I_G_ ratio) of biomass-derived carbons; Table S3: Comparative FTIR-derived functional groups and their adsorption relevance for ACPCSA5 and ACCPHSH5; Table S4: Summary of FTIR features for raw and chemically treated lignocellulosic materials; Table S5: Proposed adsorption mechanisms based on FTIR-derived functional groups; Table S6: Apparent optical bandgap energies (Eg) and corresponding electronic trends of biomass-derived carbon materials obtained from WP, PC, and CPH under different chemical activation conditions; Table S7: Nonlinear PFO and PSO kinetic parameters for COD removal from real Laguna de las Ilusiones water using raw precursors and waste-derived chemically activated carbonaceous adsorbents. The best-fit model was selected based on R^2^ and agreement with the experimental removal profile; Table S8: Comparative adsorption performance of biomass-derived and waste-derived AC adsorbents in real and simulated aqueous systems.
