# Supplementary material for: Functional Pore Accessibility and Surface Chemistry Govern Adsorption in Biomass-Derived Activated Carbons Under Real Aqueous Conditions
Source: Materials (Basel). 2026 Jun 26;19(13):2743. doi: 10.3390/ma19132743 (PMC13362594; doi:10.3390/ma19132743)
Supplement: Supplementary file 1 [file materials-19-02743-s001.zip › materials-4289897-supplementary.pdf]

SUPPLEMENTARY INFORMATION

Section S1. Adsorption Kinetics

Exploring adsorption kinetics models provides critical insights into the mechanisms that govern COD uptake onto ACPCSA5, ACCPHSH5, CPH, and PC. Investigating the adsorption rate and the time required to attain equilibrium is essential for evaluating and characterizing the adsorption performance of these materials. In this study, the experimental kinetic data were fitted to two widely used models: the Pseudo-First-Order (PFO) and Pseudo-Second-Order (PSO) kinetic models, to identify the potential rate-limiting steps and gain a better understanding of the adsorption dynamics.

The PFO kinetic model is expressed in linear form in Equation S1.

$$q_e - q_t) = ln\ ln\ q_e - k_1 t \qquad (S1)$$

The nonlinear form is given by Equation S2:

$$q_t = q_e(1 - e^{-k_1 t}) \qquad (S2)$$

where q_e (mg g^{-1}) is the amount of COD at equilibrium, q_t (mg g^{-1}) is the amount of COD adsorbed at time t (min), and k_1 (min^{-1}) is the PFO rate constant.

The PSO model in linear form is given by:

$$\frac{t}{q_t} = \frac{1}{k_2 {q_e}^2} + \frac{t}{q_e} \qquad (S3)$$

where k_2 (g mg^{-1} min^{-1}) is the PSO rate constant.

The nonlinear form of the PSO kinetic model is described by Equation S4.

$$q_t = \frac{{q_e}^2 k_2 t}{1 + q_e k_2 t} \qquad (S4)$$

where the variables are the same as in the linear form.

These models provide complementary descriptions of the adsorption kinetics. The PFO model is commonly used to describe adsorption processes dominated by physical interactions, whereas the PSO model is often associated with systems in which surface interactions or chemisorption-like contributions may be relevant. However, kinetic fitting alone does not conclusively determine the adsorption mechanism; therefore, the model assignment was interpreted together with the experimental removal profile, physicochemical characterization, and the mechanistic discussion presented in the main manuscript. The data were processed using Origin 8.5 software. By analyzing the kinetic parameters and coefficients of determination (R^2), the best-fitting model was identified, providing insight into the adsorption dynamics of the evaluated materials.

Section S2. Experimental Basis and Boundary Conditions

The techno-economic assessment was anchored to the batch adsorption conditions used in this study with real lagoon water collected from Laguna de las Ilusiones. The following boundary conditions were considered:

- Adsorbent dosage: 4 g L^{-1}, equivalent to 4 kg m^{-3}.
- Contact time: up to 180 min.
- Initial COD range: 1000–5000 mg L^{-1}.
- pH: approximately 6, corresponding to the real lagoon-water matrix.

• Operation mode: single-use adsorbent, without regeneration.

• Economic boundary: adsorbent material cost only; CAPEX and full process OPEX were excluded.

Therefore, the calculated cost per cubic meter represents a single-cycle material-use cost, not the total cost of a complete water-treatment process.

S2.1. Adsorbent Production Cost and Single-Use Treatment Cost

A medium-cost production estimate of 2.15 USD kg^{-1} was used for the waste-derived carbonaceous adsorbents. This value represents a laboratory-to-pilot screening scenario that includes the expected contribution of biomass acquisition, chemical activation, washing/neutralization, drying, handling, and indirect operational inputs. This estimate is consistent with the broader observation that, for biomass-derived AC, the economic burden is mainly associated with chemical reagents, washing, and thermal/energy-demanding steps rather than the biomass precursor itself.

The treatment cost per unit volume was estimated as:

$$C_{m^3} = D.C_{ads} \qquad (S5)$$

where D is the adsorbent dosage in kg m^{-3} and C_{ads} is the adsorbent cost in USD kg^{-1}. Under the experimental conditions:

$$C_{m^3} = 4\ kg\ m^{-3} \times 2.15\ USD\ kg^{-1} = 8.6\ USD\ m^{-3} \qquad (S6)$$

This value reflects a single-use material cost. It should not be interpreted as a complete treatment cost because adsorbent regeneration, recovery, sludge handling, labor, hydraulic operation, and infrastructure costs were not included.

S2.2. Commercial Activated Carbon Benchmark

For comparison, a commercial granular AC benchmark was included using the same adsorbent dosage applied in this study, namely 4 kg m^{-3}. Assuming a typical commercial AC price range of 5–15 USD kg^{-1}, the normalized single-use material cost would be:

$$C_{m^3} = 4\ kg\ m^{-3} \times (5-15) USD\ kg^{-1} = 20-60\ USD\ m^{-3} \qquad (S7)$$

This benchmark is not intended to represent the full cost of a commercial GAC treatment system. Rather, it provides a normalized material-cost comparison under identical dosage assumptions. In practical GAC systems, regeneration and multiple adsorption–desorption cycles can substantially reduce the effective adsorbent cost per treatment cycle. However, because regeneration was not experimentally evaluated for the waste-derived adsorbents in the present study, no regeneration credit was applied to them.

The effective adsorbent cost per cycle for a regenerable material may be expressed as:

$$C_{adscycle} = \frac{C_{ads}}{N_{eff}} + C_{regen} \qquad (S8)$$

where N_{eff} is the effective number of adsorption cycles and C_{regen} is the regeneration cost per kg of adsorbent. Since N_{eff} and C_{regen} were not experimentally determined in this work, the present techno-economic assessment conservatively assumes a single-use scenario.

S2.3. Cost Normalization by COD Removal

To compare scenarios with different influent organic loads, the economic performance was normalized by the mass of COD removed:

$$C_{COD} = \frac{C_{m^3}}{COD_0 \; x \; \eta} \quad (S9)$$

where C_{COD} is the cost per kg COD removed, COD_0 is the initial COD concentration expressed as kg m^{-3}, and η is the fractional COD removal efficiency.

For a moderate-load case of 2000 mg L^{-1} COD and 80% removal:

$$COD_{removed} = (2.0 \; kg \; m^{-3})(0.8) = 1.60 \; kg \; m^{-3} \quad (S10)$$

$$C_{COD} = \frac{8.6 \; USD \; m^{-3}}{1.60 \; kg \; m^{-3}} = 5.38 \; USD \; kg^{-1} \; COD \quad (S11)$$

For a high-load case of 5000 mg L^{-1} COD and 98% removal:

$$COD_{removed} = (5.0 \; kg \; m^{-3})(0.98) = 4.9 \; kg \; m^{-3} \quad (S12)$$

$$C_{COD} = \frac{8.6 \; USD \; m^{-3}}{4.90 \; kg \; m^{-3}} = 1.76 \; USD \; kg^{-1} \; COD \quad (S13)$$

Section S3. Additional Results of Physicochemical Characterization of Waste-Derived Activated Carbons

Table S1. XRD-derived structural parameters of biomass-derived carbons estimated using the Scherrer equation.

Sample	2θ (°)	FWHM (°)	FWHM (rad)	θ (°)	Apparent coherent domain size, D (nm)
ACPCSA5	21.09	8.07	0.1408	10.55	1.16
PC	22.14	3.92	0.0684	11.07	2.40
ACPCSH5	21.80	3.93	0.0686	10.90	2.39
ACWPSA5	21.34	4.71	0.0822	10.67	2.00
WP	21.54	4.22	0.0736	10.77	2.23
ACCPHSH5	21.12	3.82	0.0667	10.56	2.46
CPH	21.30	3.63	0.0634	10.65	2.59
ACCPHSA10	21.36	4.77	0.0833	10.68	1.97

Note: The calculated values should be interpreted as apparent coherent domain sizes rather than true crystallite dimensions due to the partially amorphous nature of biomass-derived carbons.

Table S2. Raman spectral parameters and structural disorder (I_D/I_G ratio) of biomass-derived carbons.

Sample	D band intensity (a.u.)	G band intensity (a.u.)	I_D/I_G ratio
ACWPSA5	16220.6	15457.0	1.049
ACWPSH5	6993.1	6647.8	1.052
WP	696.0	618.6	1.125

PC	3590.5	3213.7	1.117
ACPCSA5	17006.6	16687.8	1.019
ACPCSH5	37701.4	33232.3	1.134
ACCPHSA10	42972.0	43835.6	0.980
ACCPHSH5	43863.0	41942.0	1.046
CPH	47302.3	45957.5	1.029

Table S3. Comparative FTIR-derived functional groups and their adsorption relevance for ACPCSA5 and ACCPHSH5.

Functional group	Wavenumber (cm^{-1})	ACPCSA5 (Pineapple crown, H_2SO_4)	ACCPHSH5 (Cocoa Pod Husk, NaOH)	Adsorption relevance	Reference
Hydroxyl (–OH)	3332 / 3316	Strong, broad	Strong, broad	Hydrogen bonding with polar DOM	[8]
Aliphatic C–H	2920–2850	Present	Present	Structural backbone (minor role)	[64]
Carbonyl (C=O)	~1730	Strong	Weak/absent	Electrostatic interaction, polarity	[46]
Aromatic C=C (lignin)	1600–1500	Present (1512)	Present (1600, 1504)	π–π interactions with humic DOM	[67]
Carboxylate / CH_2	1450–1420	Well defined	Moderate	Surface charge contribution	[41]
Ether / alcohol (C–O–C)	1200–1000	Multiple peaks (1160, 1030)	Single dominant (1022)	Interaction with hydrophilic DOM	[72]

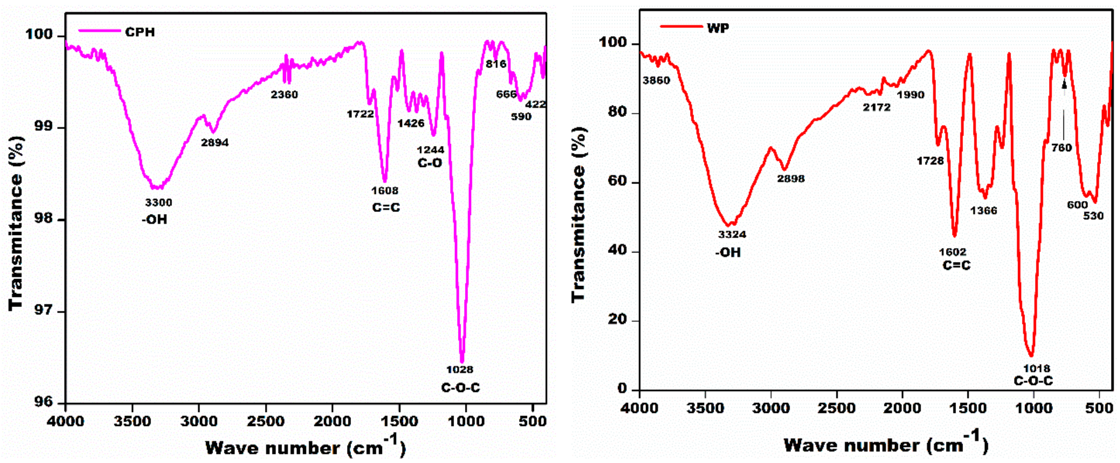

(a) (b)

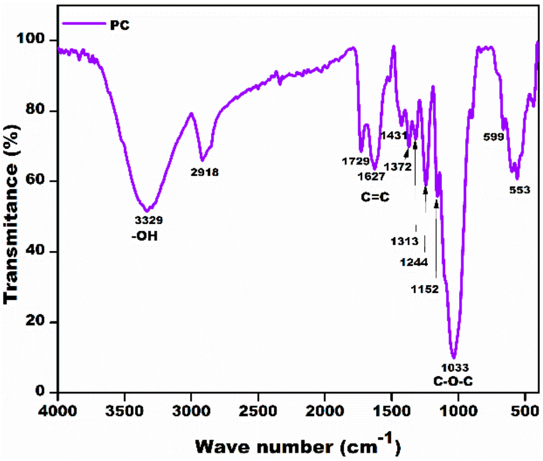

(c)

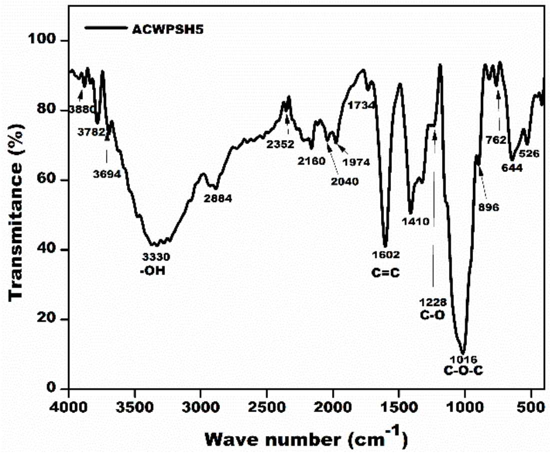

(d)

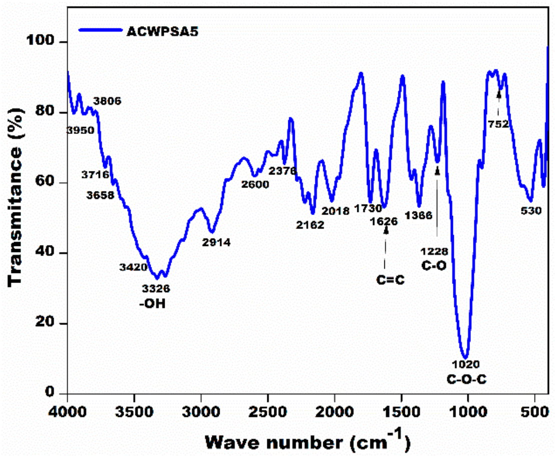

(e)

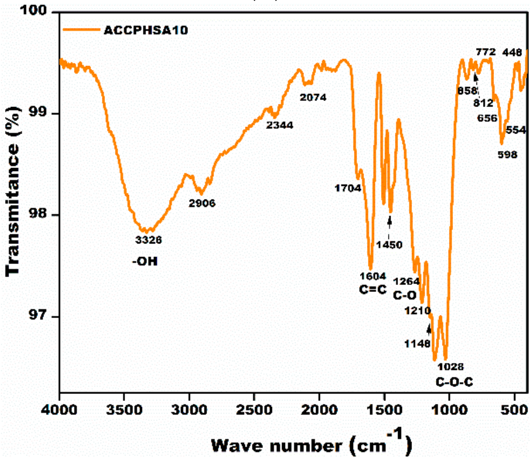

(f)

Figure S1. FTIR spectra of the raw lignocellulosic precursors, PC, WP, and CPH, and waste-derived AC derived from CPH and PC, highlighting the surface functional groups involved in adsorption processes and their potential contribution to COD removal.

Table S4. Summary of FTIR features for raw and chemically treated lignocellulosic materials.

Material	Treatment	O–H (~3300)	C=O (~1700)	Aromatic (~1600)	C–O (~1000)	Functional complexity
PC	Raw	✓	✓	✓	✓	Medium
ACPCSA5	H_2SO_4	✓✓	✓✓	✓✓	✓✓✓	**High**
ACPCSH5	NaOH	✓	✓	✓	✓✓	Medium
WP	Raw	✓	✓	✓	✓	Medium
ACWPSA5	H_2SO_4	✓✓	✓✓	✓	✓✓	Medium-high
ACWPSH5	NaOH	✓	✓	✓	✓	Medium

CPH	Raw	✓	✓	✓✓	✓	Medium
ACCPHSA10	H_2SO_4	✓✓	✓✓	✓✓	✓✓	Medium-high
ACCPHSH5	NaOH	✓	✓	✓✓	✓	**Low-medium**

Note: ✓, ✓✓, and ✓✓✓ indicate weak, moderate, and strong relative spectral contributions, respectively, based on qualitative comparison of the FTIR spectra.

Table S5. Proposed adsorption mechanisms based on FTIR-derived functional groups.

Functional group	Interaction type	DOM fraction targeted	Dominant material
–OH	Hydrogen bonding	Polar (carbohydrates, proteins)	All
–COOH / C=O	Electrostatic / complexation	Organic acids, polar DOM	ACPCSA5
Aromatic C=C	π–π interaction	Humic substances	ACCPHSH5, ACPCSA5
C–O–C	Dipole–dipole	Hydrophilic DOM	ACPCSA5

Table S6. Apparent optical bandgap energies (Eg) and corresponding electronic trends of biomass-derived carbon materials obtained from WP, PC, and CPH under different chemical activation conditions.

Material	Treatment	Eg (eV)	Electronic trend
WP	raw	2.41	insulating lignocellulosic matrix
WPSH5	NaOH	2.36	intermediate functionalization
WPSA5	H_2SO_4	2.28	increased π-conjugation
PC	raw	2.32	relatively ordered structure
ACPCSH5	NaOH	2.30	moderate π-conjugation and surface functionalization
ACPCSA5	H_2SO_4	2.15	maximum π-delocalization
CPH	raw	2.40	lignin-dominated
ACCPHSH5	NaOH	2.33	moderate modification

Section S4. Adsorption Kinetics

Table S7. Nonlinear PFO and PSO kinetic parameters for COD removal from real Laguna de las Ilusiones water using raw precursors and waste-derived chemically activated carbonaceous adsorbents. The best-fit model was selected based on R^2 and agreement with the experimental removal profile.

Adsorbent	COD_0 (mg L^{-1})	$q_{e,exp}$ (mg g^{-1})	PFO k_1 (min^{-1})	PFO R^2	PSO k_2 (g mg^{-1} min^{-1})	PSO R^2	Best-fit model
ACPCSA5	5000	1225	0.1437	0.9996	1.89 ×10-3	0.9996	PSO/PFO
ACCPHSH 5	2000	365	0.0692	0.9991	4.04×10^{-4}	0.9985	PFO
PC	5000	725	0.0035	0.8843	4.00×10^{-6}	0.8398	PFO
CPH	2000	300	0.0040	0.9435	1.10×10^{-5}	0.9095	PFO

Table S8. Comparative adsorption performance of biomass-derived and waste-derived AC adsorbents in real and simulated aqueous systems.

Precursor	Activation Method	Matrix Type	Target Parameter	Removal Efficiency (%)	Key Conditions	Reference
Coffee husk hydrochar	Hydrothermal carbonization	Synthetic (MB)	Dye removal	~95–99	pH ~6, 60–120 min	[67]
Banana stem AC	Chemical activation	Synthetic (MB)	Dye removal	>95	Mesoporous structure	[87]
Watermelon rind AC	H_2SO_4 activation	Synthetic (MB)	Dye removal	~90–99	Electrostatic + π–π	[53]
Watermelon shell magnetic AC	Chemical activation	Real wastewater	Dye removal	~99	40–60 min, pH 3–6	[23]
Cocoa shell modified adsorbent	Amine functionalization	Synthetic aqueous	Cr(VI), NO_3^-	91–99	Low concentration system	[19]
Various lignocellulosic carbons	Chemical/physical	Mixed (mostly synthetic)	Organics/ metals	80–99	Literature range	
Cocoa pod husk-derived	Acid/alkaline activation	Real lagoon matrix	COD	~70–75	pH ~6, 180 min	This study

AC (this work)						
Watermelon peel-derived AC (this work)	Acid activation	Real lagoon matrix	COD	~89–91	High COD load	This study
Pineapple crown-derived AC (ACPCSA5)	H_2SO_4 activation	Real lagoon matrix	COD	98.7 ± 1.2	Fast kinetics (~90 min)	This study

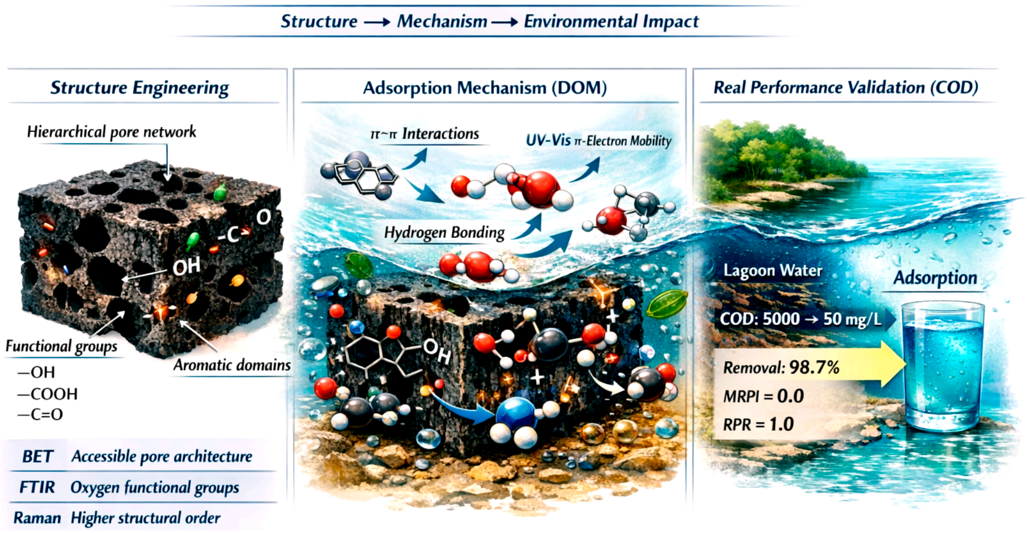

Figure S2. Conceptual schematic of the proposed adsorption mechanism based on the experimental results discussed in this study. The figure provides an illustrative summary of the structure–property–performance relationships associated with pore accessibility, surface oxygen-containing functional groups, carbon structural disorder, surface charge, and COD removal performance. It does not contain experimental micrographs, raw characterization data, or AI-generated scientific results, and should not be interpreted as direct experimental imaging.

Final Statement

The supplementary information provides full transparency of experimental data, ensuring reproducibility and supporting the interpretation of adsorption performance in complex real water matrices.
